# Proteomic Characterisation of Lupin (*Lupinus angustifolius*) Milk as Influenced by Extraction Techniques, Seed Coat and Cultivars

**DOI:** 10.3390/molecules25081782

**Published:** 2020-04-13

**Authors:** Nadia Al-Saedi, Manjree Agarwal, Wujun Ma, Shahidul Islam, Yonglin Ren

**Affiliations:** 1 College of Science, Health, Engineering and Education, Murdoch University, 90 South Street, Murdoch, WA 6150, Australia; N.Al-saedi@murdoch.edu.au (N.A.-S.); M.Agarwal@murdoch.edu.au (M.A.); 2 Department of Food Science and Biotechnology, Faculty of Agriculture, University of Baghdad, Baghdad 10071, Iraq; 3 Australia China Centre for Wheat Improvement (ACCWI), College of Science, Health, Engineering and Education, Murdoch University, 90 South Street, Murdoch, WA 6150, Australia; W.Ma@murdoch.edu.au

**Keywords:** Lupin, PBA Jurien, Mandelup, protein, two-dimensional gel electrophoresis, cheesecloth separation, centrifuge separation

## Abstract

Lupin seeds are rich in proteins and other essential ingredients that can help to improve human health. The protein contents in both whole and split seeds of two lupin cultivars (Mandleup and PBA Jurien) were used to produce the lupin milk using the cheesecloth and centrifuge method. Proteins were extracted from the lupin milk using thiourea/urea solubilization. The proteins were separated by a two-dimensional polyacrylamide gel electrophoresis and then identified with mass spectrometry. A total of 230 protein spots were identified, 60 of which showed differential abundances. The cheesecloth separation showed protein extractability much better than that of the centrifuge method for both the cultivars. The results from this study could offer guidance for future comparative analysis and identification of lupin milk protein and provide effective separation technique to determine specific proteins in the cheese-making process.

## 1. Introduction

Lupin is a grain crop that has both health and commercial value in the food industry. It is well known for its high protein and dietary fiber content. Lupin contains low starch and fat content, and the concentration of alkaloids is estimated to be below 15 mg/100 g in modern lupin cultivars, which makes it suitable for human consumption [1]. Because of the nutrient composition and high concentration of essential amino acids, which can supplement wheat to complete a balanced amino acid profile, it is regarded as a target food for healthy living [2]. Being a legume, lupin protein is a vegetable protein that has similar attributes to soybean protein [3], and it could be an effective alternative to soybean in the food industry [4,5]. The need for alternatives to animal protein has led to extensive research and breeding in protein-rich plant crops [6]. On the other hand, food manufacturers have been searching for natural, low-cost, and high-quality food ingredients, particularly sources of edible protein to tackle the increasing food demand [7]. The rising occurrences of diabetes, obesity and cardiovascular disease are also increasing the requirement for lupin-rich food products, since they have a very low glycemic index and higher protein content, and thus are beneficial to the health of consumers [8].

Lupin seed proteins are classified into several groups which have different biochemical properties that may have different potential health benefits. Its storage organs (called globulins) account for 85% of the total seed protein, with the remaining 15% forming part of the albumins [9]. The globulin includes α-, β- and γ-conglutins [10,11]. The α-conglutin falls under the 11S family and the β-conglutin is known as a 7S globulin or vicilin-like globulin [11]. ϒ-conglutin is a unique category of globulin that contains a basic monoglycosylated tetrameric element with strong links to disulphide [10]. Lupin albumins also include δ-conglutins, which belong to the family of 2S [12]. All these protein categories have different roles to play in the human body, and some are used in the manufacture of various food products with the aim of offering the required nutrition to the target consumers [13]. On the other hand, some lupin seed proteins have been identified to have allergenic effects, although only a very small percentage of people are allergic to lupin protein. The major symptoms related to lupin allergenicity include rashes and nausea, as well as anaphylaxis upon ingestion or inhalation of lupin products [14]. Accordingly, β-conglutin of *L. angustifolius* has been detected as the major allergen protein, and denoted ‘Lup an 1’ and is in the International Union of Immunological Societies (IUIS) database [14].

In addition to making bread, cookies and noodles, lupin flour can be used as an ingredient in various food types such as ice-cream, yogurt as well as plant milk. Legume milk can be used as an alternative to cow’s milk and normally contains 1.5%–3% protein. The lactic fermentation of lupin milk to produce yogurt has been well documented [15]. Thus, characterizing lupin milk proteins in food products is crucial for defining their biochemical function. The proteins of lupin flour samples were extracted in previous research works by alkaline extraction, isoelectric precipitation, salt-induced extraction and dilutive precipitation [16,17]. Since lupin milk could potentially be used for dairy products, the proteins need to be extracted by a chemical-free, water extraction process followed by separation technique. Ultrafiltration has been used as one of the separation methods in concentrating the lupin flour protein from the solid part [18]. However, sometimes ultrafiltration can change the protein profile, since it uses high pressure [19]. Furthermore, several studies have been conducted to determine the effects of mechanical treatments such as homogenisation and ultrasonic treatment on the lupin flour protein profile [20,21]. To avoid the pressure or mechanical effect on protein profile of lupin milk, the cheesecloth separation technique was also followed in this study and compared with the centrifuge technique.

Two-dimensional polyacrylamide gel electrophoresis (2D-PAGE) has been reported previously to identify proteins in lupin flour, whole lupin and whole soybean seed [14,19,22]. However, no studies were carried out to identify the proteins of lupin seed milk and how it is influenced by the presence of seed coat. Therefore, the aims of this study were to investigate the influence of two separation techniques (centrifuge and cheesecloth), and the presence of seed coat on the extractability of proteins in the milk using 2D-PAGE coupled with mass spectrometry technology. This enables the characterization of the lupin milk proteins which will improve our knowledge base to use lupin milk for direct consumption or to make cheese, yoghurt or ice cream.

## 2. Results and Discussion

### 2.1. The Protein Profile of Lupin Milk

**Figure 1:**
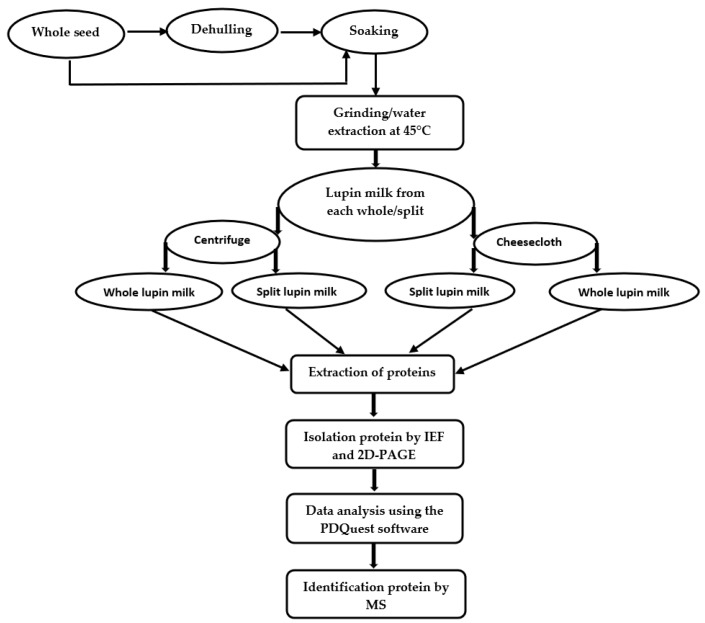
Schematic diagram showing the workflow for extraction of lupin milk and its analysis.

**Figure 2:**
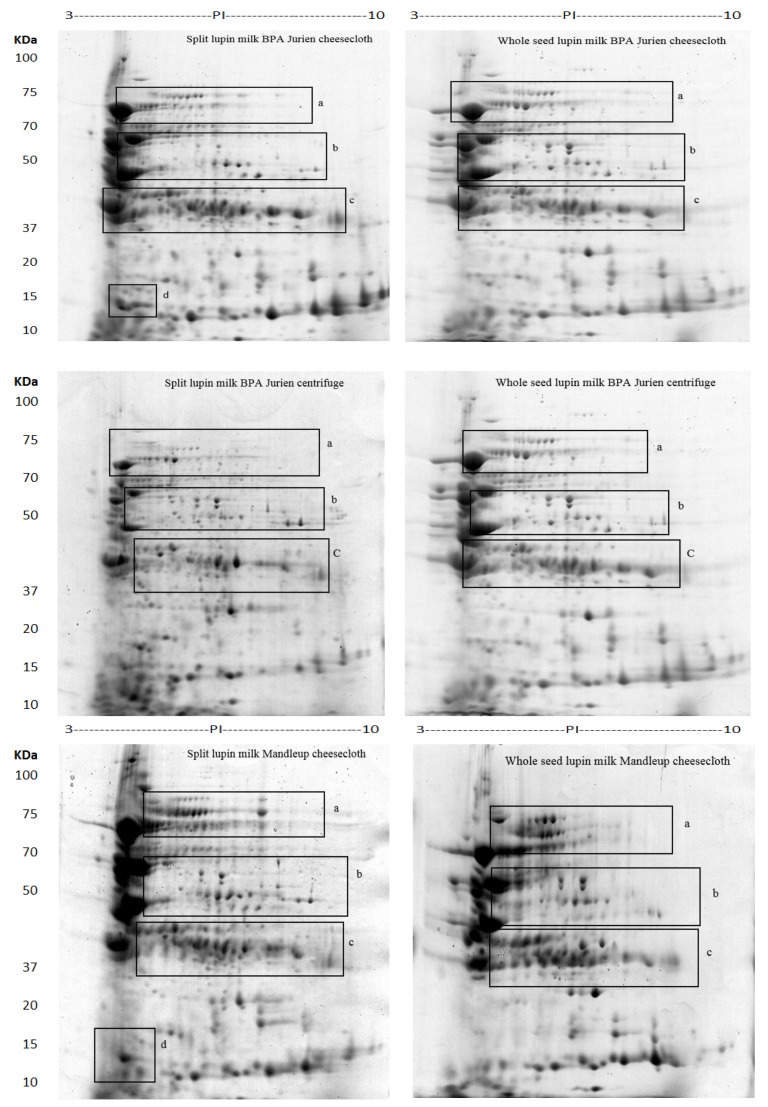

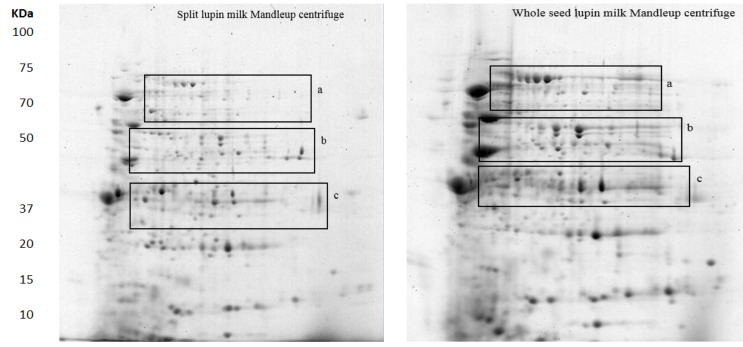
Lupin milk protein from whole seed and split lupin with a different processing profile of two cultivars of *Lupinus angustifolius* as appeared by two-dimensional gel electrophoresis signalizing overall variance of proteins in specific areas (**a**–**d**).

The extraction procedure of lupin milk and the experimental procedure of proteomic characterization are shown in Figure 1. The total protein content and numbers of protein spots identified in 2D-PAGE from lupin milk under each condition is presented in Table 1. Both the total protein content and total protein spots from cheesecloth separation were much higher than using the centrifuge separation technique. Furthermore, split lupin milk also gave a higher protein content and total protein spots than whole lupin milk. These results demonstrated successful standardisation of the 2D-PAGE procedures (Figure 2) for studying the different abundances of proteins in lupin milk. The focus was on exploring the influence of separation method, sample types and cultivars on protein extractability, which might have a potential effect on the subsequent processing such as cheese making. Using PDQuest analysis software, the standard spot number (SSP) and the quantity of each spot and standard deviation were determined, and the results are reported in Table 2. A total of 230 proteins were identified, of which 60 protein spots showed differential abundances, which were found to be either present or absent, or showed difference in protein quantity between the samples. The spot numbers, identified proteins, NCBI database accession number of the best match, molecular weight, isoelectric point, percentage sequence coverage, MOWSE score and matched peptides are listed in Table S1.

**Figure 3:**
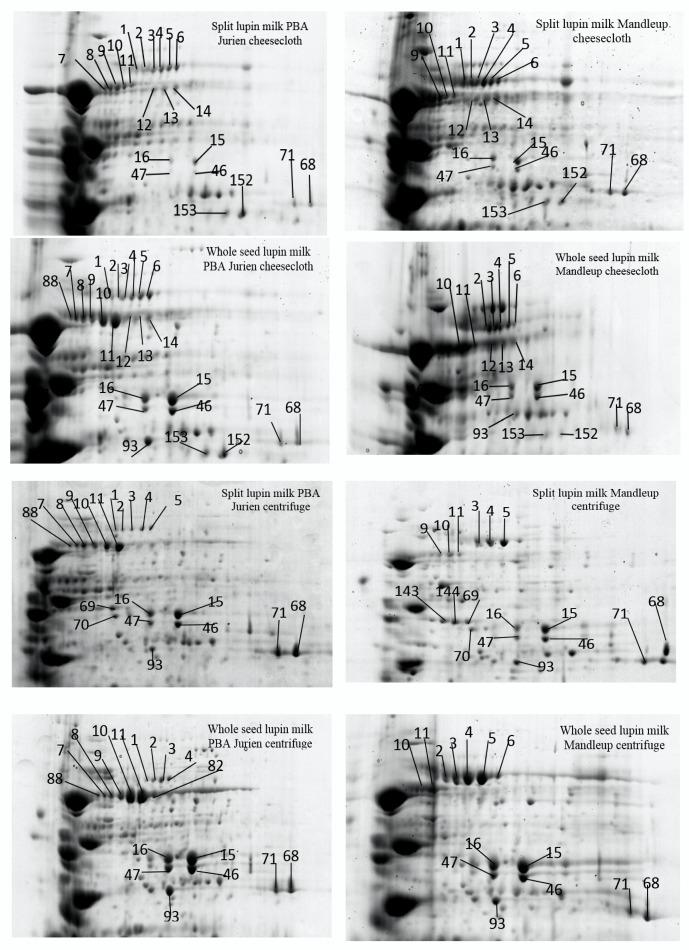
Comparison of differentiating protein abundance extractability between region a and b displayed in Figure 2 in lupin milk as influenced by seed coat and two separation techniques—cheesecloth and centrifuge—of the two cultivars.

**Figure 4:**
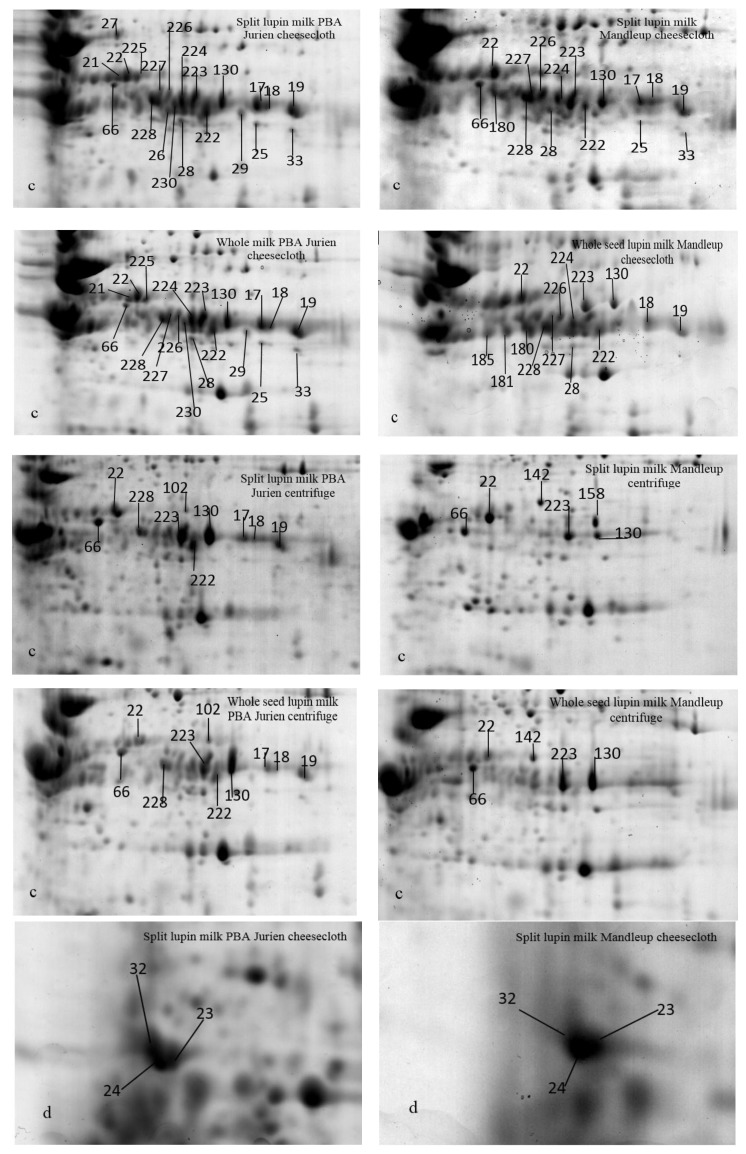
Comparison of cultivar-specific proteins of region c and d in Figure 2. Region c shows the split and whole seed lupin milk by specific separation method and region d shows the split lupin milk as influenced by cheesecloth separation of the two cultivars. There is no region d for the centrifuge separation method.

Protein resolution was relatively high in the high molecular weight region from 65 to 75 kDa, particularly in the pH range between 5 and 7. The differentially abundant proteins were positioned mainly in three specific areas of the gels, as presented in detail in Figure 2, Figure 3 and Figure 4. The most remarkable region for differentially abundant proteins was in the range of 30–35 kDa with 4–9.5 PI. In this region, twenty of the β-conglutin proteins with spot numbers of 17–22, 25, 27, 28, 29, 33, 130, 222–228 and 230 showed different levels of abundance across the separation methods and cultivars (Table 2, Figure 2 and Figure 4, Region c). Moreover, fourteen of the β-conglutins (spot numbers 1–14) showed a different level of abundance in higher molecular weight range (65–75 kDa and 5.5–6.5 PI) (Table 2 and Figure 3). However, two of the α-conglutins (spots numbers 24 and 32) and one of the β-conglutin (spot number 23) from the comparatively low molecular weight range (15–25 kDa) were found only in split lupin milk under cheesecloth separation from both cultivars with differential abundance (Table 2, Figure 2 and Figure 4, Region d). The results from this study are consistent with Foley [9], who reported that globulins were the main proteins, accounting for 85%, with the remaining 15% forming part of the albumins. The globulins consist of α-, β- and γ-conglutins. According to Magni [23], the three major proteins of the lupin seed—β-conglutin known as 7S globulin or vicilin-like globulins, α-conglutins, the 11S globulin portion, and γ-conglutins, a basic 7S protein—were present in 2D-PAGE maps.

Many factors affect the extractability of proteins, such as the presence of impurities, seed coat, and temperature. In this experiment, the extractability level of proteins was mainly affected by the presence of non-proteinaceous components such as the fibre content from the seed coat. Dietary fibre is the major component of the seed coat [24]. These non-proteinaceous components had an impact on the extractability level of proteins and quality of separation of the 2D-PAGE. Based on the results, it was evident that there was a higher number of protein spots from the lupin milk filtered with cheesecloth as compared to the centrifuge in the pH range of 5 to 7 (Figure 2 and Figure 4). For instance, the total spots detected by PDQuest Software were 231.33 from spilt lupin milk PBA Jurien cultivar in the cheesecloth separation compared to 196.33 spots in the centrifuge (Table 1).

### 2.2. Effect of the Seed Types, Separation Method and Cultivars on Extractability of Proteins in the Lupin Milk

#### 2.2.1. Seed Types

The extractability of the proteins showed large variation due to the presence of the seed coat (Table 2 and Table 3). For instance, two of the β-conglutins (spots numbers 46 and 47) and two of the α-conglutins (spots numbers 15 and 16, Figure 3) were found in higher levels of abundance in whole seed milk compared to split seed milk in both the cultivars with only centrifuge separation. As seed coat thickness and resistance are variable across the cultivars, the proteins can also be different [25]. The separation method has a significant influence on the effect of seed coat on protein extractability. For example, nine of the β-conglutins (spots numbers 18, 19, 22, 222–224 and 226–228, Table 2 and Table 3, Figure 4, Region c) had remarkably higher levels of abundances in split seed milk compared to the whole seed milk in both the cultivars, but only with cheesecloth separation. This can be attributed to the fact that in case of whole seed, the seed coat matrix impaired the separation process; as a result, the proteins were not greatly abundant in whole seeds. This could again be attributed to the fact that in centrifuge separation the seed coat being heavier in mass settled down taking with it some protein from supernatant as a result the proteins are less abundant in whole seeds. These findings are further confirmed where two of the α-conglutins (spots number 24 and 32) and one of the β-conglutins (spot 23, Table 2 and Table 3, Figure 4, Region d) were detected only in split milk for both cultivars only with cheesecloth separation. While two of the α-conglutins (spots number 69 and 70, Table 2 and Figure 3) were identified only in split seed milk in both cultivars only with centrifuge separation. Thus, we can say that seed coat can influence the protein content and the quality of lupin milk. According to Hove [26], the seed coat of three cultivars of *L. angustifolius* and one cultivar *L. albus* affected the lupin protein content. The split seed protein content was 20% higher than that of the whole seeds.

#### 2.2.2. Separation Methods

To ascertain the extractability level of proteins using two different separation techniques, a comparison was performed on lupin milk from the whole seeds and split lupin without seed coat using 2D-PAGE. The result demonstrated that a considerable number of proteins showed different extractability due to the change in separation methods (Table 1 and Table 4). However, the seed coat has significant interaction with the separation system in terms of protein extractability, and hence the influence of the separation method on the whole seed and split seed are discussed separately. In the split seeds, six β-conglutins (spot number 12–14, 21, 23 and 153) and three α-conglutins (spot numbers 24, 32 and 152) (Table 2 and Table 3, Figure 3 and Figure 4) were found only with cheesecloth separation for both cultivars.

Similarly, in the split seeds, two of the α-conglutins (spot numbers 69 and 70, Table 4 and Figure 3) were found only with centrifuge separation for both cultivars, but with a low significance of the sequence peptides and quantity of each protein’s spot, Table S1. Additionally, another eight of the β-conglutins (spots numbers 3–5, 9–11, 22, and 130) and one of the α-conglutins (spot number 66, Table 2 and Table 4, Figure 3) showed a significantly high level of abundance with the cheesecloth separation compared to centrifuge separation for both cultivars. The concentration and resolution of the protein from the centrifuged extracts was poor, and the spots were spread unevenly in a few concentrations.

Separation of protein during centrifuge in turns depends on the mass, the shape, the protein density and the movement of the molecule [27]. As a result, the α-conglutin and the β-conglutin had a different intensity when the split lupin milk was filtered with the cheesecloth (Table 2 and Figure 4). Proteins had a higher value for sequence coverage of the matched peptides (SCMP) in cheesecloth separation compared to centrifuge when they were identified by MS (Table S1). For example, the β-conglutin proteins (spot number 33 and 223 in Table S1) had 41 and 31 of the SCMP, respectively, under the cheesecloth separation, while the same protein had 8 and 10 of the SCMP, respectively, in lupin milk with centrifuge separation. This issue was found with most spots in centrifuge separation. This predicts that cheesecloth separation had a lesser effect on the protein profile of lupin milk. Hence, each peptide with the amino acid sequence was collected from lupin milk. Some peptides were matched and identified as β- and α-conglutins at a higher level of significance in the split milk cheesecloth separation. However, the same peptide’s protein was not assigned to lupin proteins β- α- and γ-conglutins in centrifuge separation. For instance, the peptides of the protein (spot number 130, Figure 4 Region c, Table S1) were identified as β-conglutins in cheesecloth separation, whereas the same protein was divided into two spots with the centrifuge method. Protein spot number 158 was identified as (Lupan Putative TAG factor protein) and another protein (spot number 130) was identified as β with a different molecular weight in centrifuge separation. This might indicate that the power of mixing by the centrifuge technique broke the peptides. In this case, the peptide ion data were not matched to possible amino acid sequences in the database. These observations are well supported by [19], which showed that high-pressure treatments affect the protein profile of *L. angustifolius* because of denaturing of the lupin protein. Another examination by [20] showed that lupin proteins are sensitive to a pressure ranging from 200 to 600 MPa, which modifies their electrostatic charge and results in changes in the structure of proteins.

On the other hand, in the case of whole seed lupin milk, one of the β-conglutins (spot number 153) and one of the α-conglutins (spot numbers 152, Table 2 and Table 4, Figure 3) were found only in cheesecloth separation for both the cultivars. Two of the α-conglutins (spots numbers 15 and 16) and one of the hypothetical proteins Tanjilg (spot number 68) (Table 2 and Table 4, Figure 3) demonstrated a higher level of abundance in centrifuge separation compared to cheesecloth for both the cultivars (Figure 3). From Table 4, it can also be observed that in centrifuge separation several β-conglutins were either absent or showed low abundance compared to the cheesecloth methods. Lupin β-conglutins have been reported as the largest allergenic protein group [14], where the majority of β-conglutin proteins (35 2D-PAGE spots out of 40) bound IgE, have the allergenic properties. Thus, combining these two observations, it can be speculated that in centrifuge separation methods the numbers of potentially allergenic protein are not coming to the lupin milk. However, only further detailed studies can confirm this.

#### 2.2.3. Cultivars

Two narrow-leaf lupin (NLL) cultivars were used: PBA Jurien and Mandleup. The cultivars of lupin showed a significant effect on the extraction of proteins in lupin milk (Table 1 and Table 5). Table 1 shows that lupin milk from PBA Jurian, irrespective of seeds coat and separation method, demonstrated higher total protein content and a greater number of protein spots in comparison to Mandelup. Five of the β -conglutins (spots numbers 10, 11, 22 130 and 223, Figure 3 and Figure 4, Table 2 and Table 5) showed a higher level of abundance in PBA Jurien compared to Mandelup, irrespective of separation techniques or seed coat. In contrast, the separation techniques had a considerable influence on the protein extractability of both cultivars. For instance, six of the β-conglutins (spot numbers 7, 8, 21, 29, 225 and 230, Table 2 and Table 5) were recognized in both split and whole milk seed of PBA Jurien cultivar in cheesecloth separation. Meanwhile, one of the β-conglutin proteins (spot number 180, Table 2 and Table 5) was found in both split and whole milk seed of Mandelup cultivar in cheesecloth separation. Additionally, nine of the β-conglutins (spot numbers 1, 7, 8, 17–19, 88, 102 and 228, Figure 3 and Figure 4, Table 2 and Table 5) were detected in both split and whole seed milk of PBA Jurien in centrifuge separation. On the other hand, the Mandelup cultivar had one of α-conglutins (spot number 142, Figure 3).

Twenty-two of the β-conglutins (spots numbers 2–6, 9–14, 18,19, 22, 28, 130, 222–224 and 226–228, Figure 3 and Figure 4) were shown in an extraordinarily higher level of abundance in the split and whole milk seed of PBA Jurien with the cheesecloth separation compared to split and whole seed of Mandelup cultivar. According to the study by Islam [28], nineteen of the β-conglutin and eight of the allergenic proteins were detected with different expression in the four narrow-leafed lupin cultivars Uniharvest, Yorrel, Tanjil and Coromup showing that the genetic composition and gene content in the sequences varied. Regardless of cultivars, the main protein in most cultivars of lupin seeds was β-conglutin [29], which was found in our study too. As lupin has recently been recognized as a human health food, more and more cultivars with different genome assemblies and gene contents are being developed [30], and this in turn can affect the pattern of protein sequences. From our current study and previous research, it is evident that the composition of the protein sequences depicts the orientation of the genomic patterns and sequences.

## 3. Materials and Methods

### 3.1. Chemicals

Chemicals for electrophoresis, including sodium dodecyl sulfate (SDS), *N*,*N*,*N*_,*N*_-tetramethylethylendiamine (TEMED), ammonium persulfate, thiourea, urea, dithiothreitol (DTT), CHAPS, glycerol, agarose, bromophenol blue, Coomassie Brilliant Blue, iodoacetamide and Tris–HCl (pH 8.8), were purchased from Sigma (Willettion, WA, Australia). From Bio-Rad (Gladesville, New South Wales, Australia) 40 % Acrylamide/bis solution ampholytes (pH 3–10) and 17 cm IPG strips with pH 3–10 catalogue # 163–2009 were purchased. All chemicals above were standard laboratory grade chemicals. Water from Millipore, Bedford reverse osmosis system (Burlington, MA, USA) was used for making all solutions.

### 3.2. Preparation of Lupin Samples and Lupin Milk

Two Australian sweet lupin (*Lupinus angustifolius*) cultivars, Pulse Breeding Australia -PBA Jurien and Mandelup were selected. They are newly developed disease resistant cultivars. The cultivar PBA Jurien was obtained from Eastern Districts Seed Cleaning Company (Kellerberrin, Western Australia) and the other cultivar Mandelup was sourced from Department of Primary Industries and Regional Development (DPIRD), Western Australia. The tested lupin samples were newly harvested (2017/2018) pesticide free seeds and stored at −20 °C until use.

For preparation of lupin milk, both the whole seeds with seed coat (hulls) and split seeds without seed coat of two cultivars (PBA Jurien and Mandelup), 10 gms each were taken. Two different separation methods: cheesecloth and centrifugation were used to extract milk. For preparation of split seeds, the seeds were broken into halves and seed coat removed with the mortar and pestle.

Each 10 g of dry half split lupin and whole seed were soaked separately in water for overnight with the ratio of 1:3 (lupin:water) at room temperature (24 ± 1 °C). A stainless-steel gas-tight blender (250 mL), fitted with a screw top lid containing a septum, was used for the grinding of soaked samples. Soaked whole seed (10 g) were placed in a blender containing 100 mL of water maintained at a temperature of 45 °C. The sample was grinded for 5 min, and the mixture was divided into two equal parts. One portion was filtered using four layers of cheesecloth, and the other fraction was filtered using a centrifuge from AIPU, Hangzhou, China at 2600× *g* for 5 min. The filtrate was stirred to get the final volume of lupin milk. Using the same procedure, the milk was prepared three times from three different lot of seeds. The workflow diagram is depicted in Figure 1.

### 3.3. Total Proteins

AOAC (2000) methods were used to determine the protein (N × 5.7) contents (method 981.10C) [31].

### 3.4. Extraction of Proteins

Milk from cheesecloth and centrifuge separation was used for extraction of proteins. The protein was precipitated by incubating 400 µL of the lupin milk with 1600 µL of ice cold acetone at −20 °C overnight. The precipitate was collected by centrifugation at 13,000× *g* for 10 min and discarding supernatant. The protein pellet was dissolved in rehydration buffer (7M urea, 2M thiourea, 4% CHAPS, 65 mM DTT and 2% IPG buffer). The samples were incubated for 4-5 h at room temperature. Protein concentration was determined by using RC DC protein assay kit (Bio-Rad, Herculles, CA) and Lambda 25 UV–vis spectrometer (PerkinElmer). From Based on the calibration curve, 900 µg of lupin milk protein was loaded onto IPG strips for each sample.

### 3.5. Separation of Proteins

The proteins were separated by Iso-electric focusing (IEF) and were carried on 17 cm IPG strips with pH 3–10 which were rehydrated with the buffer (7 M urea, 2 M thiourea, 2% CHAPS, 65 mM DTT and 2% IPG buffer) containing 900 µg of protein. The strips were focussed at 250 V for 1 h, 1000 V for 1 h, 10,000 V for 5 h, 70,000 V for 1 h and 500 V for 48 h at 20 °C using Protein IEF cell (BioRad). The gel strips were incubated with equilibration buffer [50 mM Tris–HCl (pH 8.8), 6 M urea, 30% (*v*/*v*) glycerol, 2% (*w*/*v*) SDS and 0.002% bromophenol blue, containing 65 mM DTT] for 15 min and another 10 min by replacing DTT with 135 mM iodoacetamide in the same buffer and subsequently placed onto 12% acrylamide/bis (31.5:1) gels, using Protean II Xi cell (Bio-Rad). Strips were overlayed with agarose sealing solution (1% agarose and 0.002% bromophenol) and running buffer consisted of 2.5 mM Tris–Base, 19.2 mM glycine and 0.01% SDS. The 2D-PAGE gels were visualized by staining with Coomassie Brilliant Blue (CBB). Three biological replications were run three times with individual extraction and IEF.

### 3.6. Data Analysis

The comparative analysis of the 2-DE gels was performed using the PDQuest software. The spots were detected by automatic spot detection; gel images were carefully edited. Before spot matching, one of the gel images was selected as the reference gel or a master gel that includes all essential information of the protein ingredients in different gels. The data from image analysis were transferred to PDQuest software for recognizing protein spots, which show quantitative variations based on intensity with a unique standard spot number SSP to provide location of the spot. Statistical analysis of the data was carried out using Microsoft Excel 365, 2019 than the quantity and standard deviation (Sd) were calculated from three spots in different gels.

### 3.7. Identification of Protein

Protein spots were resected from Coomassie Brilliant Blue stained two-dimensional gels and analysed further by mass spectrometric peptide sequencing. The spots were analysed by Proteomics International Ltd. Pty, UWA, Perth, Australia. Protein samples were digested with trypsin and the peptides were extracted according to standard techniques [32]. Peptides were analysed by LC-MS using the Agilent 1260 infinity HPLC system coupled to Agilent 1260 Chipcube Nanospray interface on an Agilent 6540 mass spectrometer. Tryptic peptides were loaded onto a ProtID-Chip-150 C18 column (Agilent) and separated with a linear gradient of water/acetonitrile/0.1% formic acid (*v*/*v*). The software Mascot (Matrix Science) with taxonomy set to Viridiplantae (Green Plants) was analysed to identify the proteins. The search parameters for LC-MS/MS on the Agilent 6540 mass spectrometer were as peptide tolerance of ±0.2. The peptide charges were set at 2+3+ and 4+ and 1 missed cleavage, the significance threshold at *P* < 0.05. Generally, a match was accepted where two or more peptides from the same protein were present in a protein entry in the Viridiplantae database. Protein identification was completed by searching the National Centre for Biotechnology Information (NCBI) nonredundant database using the Mascot search engine.

## 4. Conclusions

Increasing interest in lupin protein as an alternative to animal and soybean proteins in producing future lupin-based dairy products initiated the need for this research. The two separation methods were used to test the extractability of the whole seed and split seed lupin milk protein from different cultivars. The cheesecloth extraction approach is more suitable than the centrifuge method for the recovery of the lupin milk protein in both the whole seed and the split seed lupin milk from both cultivars. The cheesecloth separation used in this work allowed the detection and identification of two of the α-conglutins and one of the β-conglutins only in split milk for both cultivars. This report confirmed that Cultivar PBA Jurien contains more protein in comparison with cultivar Mandelup, for instance, twenty-two of the β-conglutins showed significantly higher levels of abundance in the split and whole milk seed of PBA Jurien with the cheesecloth separation compared to split and the whole seed of Mandelup cultivar.

The overall effects of the separation method on the protein profile of the processed lupin milk from whole seeds and spilt seeds have never been considered so far. Future studies of processing dairy products will be benefitted from this proteomic reference map of lupin milk and will help in understanding the specific proteins that could be responsible for the coagulation of lupin milk and for creating lupin cheese and yogurt.

## Figures and Tables

**Table 1:** List of the total protein content and numbers of protein spots detected by PDQuest software from 2DGE gels of lupin milk of each condition.

Cultivars	Separation Method	Type of Lupin Seeds	Total Protein (g/L) Mean ± SD (*n* = 3)	Spots Numbers Mean ± SD (*n* = 3)
PBA Jurien	cheesecloth	Split	27.53 ± 1.84	231.33 ± 1.15
		Whole	20.98 ± 1.27	201 ± 1
	centrifuge	Split	20.49 ± 1.51	196.33 ± 1.52
		Whole	14.49 ± 1.16	158.33 ± 0.57
Mandelup	cheesecloth	Split	27 ± 2.03	204 ± 1
		Whole	20.38 ± 1.92	190.67 ± 0.57
	centrifuge	Split	19.85 ± 1.17	189.33 ± 0.57
		Whole	14.94 ± 1.55	180 ± 1

SD = standard deviation; Number of replicates (*n* = 3.).

**Table 2:** Quantitative list of each identified protein spot with respect to extractability in the lupin milk made from different samples types, separation technique and the cultivar. The spots are significantly different (*p* < 0.05) at PDQuest Bio-Rad.

		Split Lupin Milk PBA Jurien		Whole Lupin Milk PBA Jurien		Split Lupin Milk Mandelup		Whole Lupin Milk Mandelup	
		Cheesecloth	Centrifuge	Cheesecloth	Centrifuge	Cheesecloth	Centrifuge	Cheesecloth	Centrifuge
Spot No	SSP	Mean ± SD (*n* = 3)	Mean ± SD (*n* = 3)	Mean ± SD (*n* = 3)	Mean ± SD (*n* = 3)	Mean ± SD (*n* = 3)	Mean ± SD (*n* = 3)	Mean ± SD (*n* = 3)	Mean ± SD (*n* = 3)
1	4802	51 ± 1.41	29.53 ± 2.71	62.14 ± 1.16	20.75 ± 0.87	41.50 ± 2.67	ND	ND	ND
2	4803	56.69 ± 2.66	39.49 ± 1.17	75.29 ± 4.20	30.69 ± 1.12	43.89 ± 3.10	ND	38.19 ± 2.03	35.44 ± 1.44
3	5802	61.59 ± 3.60	27.95 ± 1.26	78.22 ± 1.40	22.03 ± 2.35	47.47 ± 2.06	33.60 ± 1.32	35.17 ± 0.57	30.61 ± 0.74
4	5804	92.63 ± 1.91	33.39 ± 1.10	104.57 ± 3.69	31.55 ± 2.15	67.07 ± 1.8	41.66 ± 1.23	52.95 ± 4.74	44.95 ± 0.95
5	5806	78.74 ± 1.22	47.74 ± 2.51	84.34 ± 2.95	ND	61.30 ± 0.61	50.95 ± 0.26	58.15 ± 0.58	55.38 ± 0.40
6	5808	79.40 ± 1.34	ND	81.45 ± 0.25	ND	71.63 ± 2.59	ND	53.62 ± 2.83	20.72 ± 1.18
7	2802	98.30 ± 0.84	60.37 ± 1.44	108.13 ± 4.50	44.48 ± 0.99	ND	ND	ND	ND
8	2803	113.25 ± 4.23	64.72 ± 1.13	160.5 ± 2.80	55.63 ± 0.75	ND	ND	ND	ND
9	3801	114.49 ± 5.20	37.48 ± 1.77	163.79 ± 3.75	26.1 ± 2.19	44.88 ± 2.99	21.06 ± 0.82	ND	ND
10	3802	147.59 ± 2.82	66.33 ± 1.52	155.21 ± 2.24	49.15 ± 2.73	83.81 ± 1.05	37.16 ± 0.76	52.77 ± 1.83	43.68 ± 2.36
11	3804	85.27 ± 1.48	74.63 ± 2.90	260.20 ± 3.63	73.24 ± 1.73	59.43 ± 2.95	23.86 ± 3.5	33.56 ± 0.58	30.53 ± 0.93
12	5801	46.01 ± 3.36	ND	30.57 ± 1.12	ND	30.59 ± 2.28	ND	21.25 ± 0.65	ND
13	5804	54.53 ± 1.60	ND	33.19 ± 2.34	ND	36.06 ± 0.55	ND	30 ± 1.12	ND
14	5807	61.05 ± 1.59	ND	38.47 ± 1.43	ND	41.77 ± 0.43	ND	33.11 ± 1.11	ND
15	6506	166.15 ± 5.16	251.43 ± 1.68	240.37 ± 3.75	266.77 ± 3.16	194.52 ± 3.84	206.84 ± 1.90	227.61 ± 4.49	238.54 ± 5.21
16	6505	130.97 ± 6.40	221.05 ± 1.79	238.83 ± 2.63	298.11 ± 1.78	191.46 ± 2.87	214.00 ± 1.55	215.04 ± 2.03	247.86 ± 5.04
17	6306	501.68 ± 5.11	54.85 ± 0.89	254.56 ± 2.98	32.66 ± 1.22	232.86 ± 3.69	ND	ND	ND
18	7304	393.13 ± 4.70	33.71 ± 2.83	311.65 ± 2.43	23.66 ± 1.79	160.93 ± 3.79	ND	76.18 ± 2.46	ND
19	8301	407.24 ± 3.58	40.51 ± 0.78	365.69 ± 0.90	20.76 ± 1.24	195.39 ± 3.92	ND	79.51 ± 0.53	ND
21	3405	567.09 ± 3.26	ND	105.35 ± 2.07	ND	ND	ND	ND	ND
22	4401	218.94 ± 4.51	135.26 ± 2.64	204.80 ± 3.82	46.16 ± 5.01	196.97 ± 2.20	102.37 ± 2.44	178.05 ± 0.15	25.53 ± 1.06
23	1208	417.46 ± 2.86	ND	ND	ND	548.56 ± 5.12	ND	ND	ND
24	1210	314.10 ± 1.80	ND	ND	ND	415.90 ± 6.44	ND	ND	ND
25	7204	78.68 ± 3.2	ND	49.85 ± 3.2	ND	40.72 ± 2.27	ND	ND	ND
26	5307	73.65 ± 0.68	ND	ND	ND	ND	ND	ND	ND
27	3404	178.12 ± 5.24	ND	61.05 ± 1.58	ND	ND	ND	ND	ND
28	5202	107.12 ± 4.67	ND	95.07 ± 4.79	ND	64.06 ± 3.56	ND	44.42 ± 1.42	ND
29	7201	145.77 ± 4.39	ND	126.81 ± 5.21	ND	ND	ND	ND	ND
32	1205	381.31 ± 3.20	ND	ND	ND	459.90 ± 5.21	ND	ND	ND
33	8201	92.23 ± 1.51	ND	71.89 ± 4.06	ND	30.04 ± 0.39	ND	ND	ND
46	6502	44.85 ± 0.70	183.11 ± 5.18	142.16 ± 3.93	190.45 ± 1.31	77.36 ± 1.04	95.33 ± 3.50	110.68 ± 2.30	132.66 ± 4.02
47	5502	21.88 ± 1.65	206.73 ± 3.82	178.29 ± 1.28	290.27 ± 0.81	80.54 ± 2.35	86.15 ± 1.81	98.30 ± 1.41	121.00 ± 1.00
66	3301	121.76 ± 0.92	76.06 ± 1.42	97.43 ± 4.20	62.81 ± 1.13	79.88 ± 0.58	74.25 ± 3.54	ND	65.79 ± 2.87
68	8501	50.81 ± 2.04	70.65 ± 3.52	76.39 ± 2.18	88.83 ± 1.35	81.82 ± 5.14	91.38 ± 3.85	30.55 ± 0.58	45.81 ± 1.57
69	5607	ND	21.59 ± 1.16	ND	ND	ND	18.09 ± 0.58	ND	ND
70	5507	ND	31.08 ± 1.13	ND	ND	ND	14.13 ± 0.80	ND	ND
71	8401	42.92 ± 2.68	78.65 ± 0.68	80.15 ± 2.60	89.99 ± 1.59	63.51 ± 3.84	82.74 ± 2.42	40.54 ± 0.49	68.21 ± 0.80
82	5801	ND	ND	ND	44.03 ± 0.71	ND	ND	ND	ND
88	2802	ND	22.39 ± 0.93	54.77 ± 1.39	33.63 ± 0.75	ND	ND	ND	ND
93	5401	ND	89.53 ± 1.05	136.34 ± 1.05	115.99 ± 0.73	ND	104.13 ± 1.88	34.51 ± 0.58	126.40 ± 2.12
102	6404	ND	21.97 ± 0.29	ND	32.94 ± 0.65	ND	ND	ND	ND
130	6306	549.00 ± 5.08	236.34 ± 1.05	265.33 ± 1.57	188.33 ± 2.54	355.63 ± 3.82	198.38 ± 1.51	167.82 ± 3.14	132.37 ± 3.20
142	7404	ND	ND	ND	ND	ND	81.11 ± 0.90	ND	74.04 ± 0.30
143	4505	ND	ND	ND	ND	ND	16.91 ± 0.77	ND	ND
144	4612	ND	ND	ND	ND	ND	18.58 ± 0.59	ND	ND
152	7401	190.41 ± 1.85	ND	179.77 ± 2.78	ND	98.466 ± 2.56	ND	50.31 ± 0.69	ND
153	6401	106.60 ± 3.01	ND	97.89 ± 1.75	ND	87.30 ± 2.99	ND	68.88 ± 0.39	ND
180	4303	ND	ND	ND	ND	85.41 ± 0.74	ND	72.82 ± 0.58	ND
181	3302	ND	ND	ND	ND	ND	ND	87.63 ± 1.19	ND
185	3406	ND	ND	ND	ND	ND	ND	90.88 ± 0.28	ND
222	6303	672.66 ± 2.64	230.70 ± 1.33	569.39 ± 5.81	189.54 ± 2.42	433.7 ± 2.31	ND	75.86 ± 0.81	ND
223	6301	431.56 ± 2.73	209.31 ± 3.40	388.92 ± 5.48	198.01 ± 1.95	324.37 ± 4.14	178.60 ± 1.09	235.81 ± 1.19	162.45 ± 0.64
224	5305	470.67 ± 1.28	ND	311.70 ± 4.91	ND	281.78 ± 5.33	ND	182.23 ± 2.16	ND
225	4402	201.33 ± 3.50	ND	150.53 ± 4.40	ND	ND	ND	ND	ND
226	5303	191.00 ± 4.16	ND	83.65 ± 5.11	ND	75.71 ± 2.47	ND	64.54 ± 0.82	ND
227	5302	146.33 ± 1.52	ND	104.45 ± 3.39	ND	69.81 ± 2.68	ND	35.188 ± 2.94	ND
228	5301	206.23 ± 1.66	42.07 ± 1.86	196.74 ± 5.00	34.49 ± 0.76	157.35 ± 2.93	ND	86.32 ± 3.96	ND
230	5304	581.00 ± 5.73	ND	354.70 ± 2.26	ND	ND	ND	ND	ND

**Note:** Protein spots with abundance differences between different samples are listed in this table. SSP = standard spot number; SD = standard deviation; Number of replicates (*n* = 3); ND = not detected.

**Table 3:** List of the lupin proteins with respect to extractability in the lupin milk as influenced by seed coat.

Cultivars	Separation Method	Type of Lupin Seeds to Make Milk	Protein Spots Present	Higher Level of Abundance
PBA Jurien	cheesecloth	Split	23, 26 [β-conglutins] 24, 32 [α-conglutins]	12–14, 17–19, 21, 22, 25, 27–29, 33 130, 153, 222–228, 230 [β-conglutins] 152, 66 [α-conglutins]
		Whole	93 [α-conglutins] 88 [β-conglutins]	1–11, 46, 47, 71 [β-conglutins] 15, 16 [α-conglutins] 68 [hypothetical protein Tanjilg]
PBA Jurien	centrifuge	Split	69, 70 [α-conglutins] 5 [β-conglutins]	1–4, 7–11, 17–19, 22, 130, 222, 223, 228 [β-conglutins] 66 [α-conglutins]
		Whole	82 [Lupan Putative TAG factor protein]	15, 16, 93 [α-conglutins] 46, 47, 71, 88, 102 [β-conglutins] 68 [hypothetical protein Tanjilg]
Mandelup	cheesecloth	Split	1, 9, 23, 17, 25, 33, 66 [β-conglutins] 24, 32 [α-conglutins]	2–6, 10–14, 18, 19, 22, 28, 71, 222–224, 226–228 130, 153, 180 [β-conglutins] 152 [α-conglutins] 68 [hypothetical protein Tanjilg]
		Whole	185, 93 [α-conglutins] 181 [β-conglutins]	15, 16 [α-conglutins] 46, 47 [β-conglutins]
Mandelup	centrifuge	Split	69, 70, 143, 144 [α-conglutins] 9 [β-conglutins] 158 [Lupan Putative TAG factor protein]	66, 142 [α-conglutins] 22, 71, 130, 223 [β-conglutins] 68 [hypothetical protein Tanjilg]
		Whole	2, 6 [β-conglutins]	15, 16, 93 [α-conglutins] 3–5, 10, 11, 46, 47 [β-conglutins]

**Note:** Only protein spots with abundance differences in comparison of seed coat are presented in this table.

**Table 4:** List of the lupin proteins compared between cheesecloth and centrifuge filtration with respect to protein spots and their identification.

Lupin Milk	Separation Method	Protein Spots Present	Higher Level of Abundance
Split seed lupin milk PBA Jurien	Cheesecloth	6, 12–14, 21, 23, 25–29, 33, 153, 224–227, 230 [β-conglutins] 24, 32, 152 [α-conglutins]	1–5, 7–11, 17–19, 22, 130, 222, 223, 228 [β-conglutins] 66 [α-conglutins]
	Centrifuge	88, 93, 102 [β-congutins] 69, 70 [α-conglutins]	15, 16 [α-congutins] 46, 47, 71 [β-congutins] 68 [hypothetical protein Tanjilg]
Whole seed lupin milk PBA Jurien	Cheesecloth	5, 6, 12–14, 21, 25, 28, 29, 33, 153, 224–227, 230 [β-congutins] 152 [α-conglutins]	1–4,7–11, 17–19, 22, 88, 130, 222, 223, 228 [β-congutins] 66, 93 [α-conglutins]
	Centrifuge	102 [β-conglutins] 82 [Lupan Putative TAG factor protein]	15, 16 [α-conglutins] 46, 47, 71 [β-conglutins] 68 [hypothetical protein Tanjilg]
Split seed lupin milk Mandelup	Cheesecloth	1, 2, 6, 12–14, 17–19, 23, 25, 28, 33, 153, 180, 222, 224, 226–228 [β-conglutins] 24, 32, 152 [α-conglutins]	3-5, 9–11, 22, 130, 223 [β-conglutins] 66 [α-conglutins]
	Centrifuge	69, 70, 93, 142–144 [α-conglutins] 158 [Lupan Putative TAG factor protein]	46, 47, 71 [β-congutins] 16, 15 [α-conglutins] 68 [hypothetical protein Tanjilg]
Whole seed lupin milk Mandelup	Cheesecloth	12–14,18, 19, 28, 153, 180, 181, 222, 224, 226–228 [β-conglutins] 152, 185 [α-conglutins]	2–6, 10, 11, 22,130, 223 [β-conglutins]
	Centrifuge	142, 66 [α-conglutins]	46, 47, 71 [β-conglutins] 15, 16, 93 [α-conglutins] 68 [hypothetical protein Tanjilg]

**Note:** Only protein spots with abundance differences in comparison of separation techniques are presented in this table.

**Table 5:** List of the lupin proteins compared between PBA Jurien and Mandelup cultivars with respect to protein spots and their identification.

Lupin Milk	Cultivars	Proteins Spots Present	Higher Level of Abundance
Split seed lupin milk cheesecloth separation	PBA Jurien	7, 8, 21, 26, 29, 225, 230 [β-conglutins]	1–6, 9–14, 17–19, 22, 25, 28, 33, 130, 153, 222–224, 226–228 [β-conglutins] 152 [α-conglutins]
	Mandelup	180 [β-conglutins]	15, 16, 24, 32 [α-conglutins] 23, 46, 47, 71 [β-conglutins] 68 [hypothetical protein Tanjilg]
Whole seed lupin milk cheesecloth separation	PBA Jurien	1, 7–9, 17, 21, 25, 29, 33, 88, 225, 230 [β-conglutins] 66 [α-conglutins]	15, 16, 152, 93 [α-conglutins] 2–6, 10–14, 18, 19, 22, 28, 46, 47, 130,153, 222–224, 226–228 [β-conglutins]
	Mandelup	180, 181 [β-conglutins] 185 [α-conglutins]	NPS
Split seed lupin milk centrifuge separation	PBA Jurien	1, 2, 7, 8, 17-19, 88, 102, 222, 228 [β-conglutins]	15, 16, 70, 66, 69 [α-conglutins] 9–11, 22, 46, 47, 130, 223 [β-conglutins]
	Mandelup	143, 144, 142 [α-conglutins] 158 [Lupan Putative TAG factor protein]	3–5, 71 [β-conglutins] 93 [α-conglutins] 68 [hypothetical protein Tanjilg]
Whole seed lupin milk centrifuge separation	PBA Jurien	1, 7–9, 17–19, 88, 102, 228 [β-conglutins] 82 [Lupan Putative TAG factor protein]	15, 16 [α-conglutins] 10, 11, 22, 46, 47, 71, 130, 223 [β-conglutins] 68 [hypothetical protein Tanjilg]
	Mandelup	142 [α-conglutins] 5, 6 [β-conglutins]	2–4 [β-conglutins] 93, 66 [α-conglutins]

**Note:** Only protein spots with abundance differences in comparison of cultivars are presented in this table. NPS: no protein spot.

## Supplementary Materials

The following are available online at https://www.mdpi.com/1420-3049/25/8/1782/s1, Table S1. MS/MS identification of differentiating proteins between two cultivars. The Matching has been achieved using Mascot sequence matching software (Matrix Science) with the taxonomy set to Viridiplanate (Green Plants). The spots are significantly different (*p* < 0.05) at PDQuest Bio-Rad.
