# Supplementary material for: Proteomic Characterisation of Lupin (Lupinus angustifolius) Milk as Influenced by Extraction Techniques, Seed Coat and Cultivars
Source: Molecules. 2020 Apr 13;25(8):1782. doi: 10.3390/molecules25081782 (PMC7221801; doi:10.3390/molecules25081782)
Supplement: Supplementary file 1 [file molecules-25-01782-s001.pdf]

Table S1. MS/MS identification of differentiating proteins between two cultivars. The matching has been achieved using Mascot sequence-matching software (Matrix Science) with the taxonomy set to Viridiplanate (Green Plants). The spots are significantly different (P<0.05) at PDQuest Bio-Rad.

No	Protein	NCBI accession number	Database Theoretical MW/PI	Sequence coverage (%)	MOWES Score	Peptides
1	β-Conglutin (*Lupinus angustifolius)*	gi|1117364351	75163/5.75	37	1071	LQNYRIVEFQ; EYGDALRLPA; QQSYFSGFSK; EGVIVRVSKE; LLGFGINADE; QQSYFANAQP; HRLRNPYYFS; SRPNTLILPK; NTLEATFNTH; QIQELRKHAQ; QQDEQEVEEV; NQRNFLAGSE; QQQQQQREKE; SERFQTRYKN; HSDADYILVV; DNQDLRVVKL; YEEIQRILLG; SSSRKGKPSE; RSYNARLSEG; DNVIRQLDRE; LNGRATITIV; AIPINNPGKF; YEDEQEDEEQ; SGPFNLRSNE; VKELIFPGSA; FDQRTNRLEN; NPDKRQAYNL; YDFYPSRTKD; RREQEQSHQD; EDVERLIRNQ
2	β-Conglutin (*Lupinus angustifolius)*	gi|1117364351	75163/5.75	40	1407	LQNYRIVEFQ; EYGDALRLPA; QQSYFSGFSK; EGVIVRVSKE; ELVGIRNQQR; LLGFGINADE; HRLRNPYYFS; SRPNTLILPK; NTLEATFNTH; QIQELRKHAQ; QQDEQEVEEV; NQRNFLAGSE; HSDADYILVV; YEEIQRILLG; SSSRKGKPSE; RSYNARLSEG; LNGRATITIV; YEDEQEDEEQ; SGPFNLRSNE; FDQRTNRLEN; YDFYPSRTKD; RREQEQSHQD; PIYSNKFGNF; EDVERLIRNQ
3	β-Conglutin (*Lupinus angustifolius)*	gi|1117364351	75163/5.75	42	1420	NPDKRQAYNL; YDFYPSSTKD; LQNYRIVEFQ; NQRNFLAGSE; AIPINNPGNF; LNGRATITIV; DNQNLRVVKL; YEEIQRIILG; SSSGKGKPSE; DNVIRQLDRE; FDQRTNRLEN; PIYSNKFGNF; EDVERLIRNQ; HSDADYILVV; QQSYFSGFSK; EGVIVRVSKE; ELVGIRNQQR; LLGFGINADE; QQSYFANAQP; HRLRNPYYFS; SRPNTLILPK; NTLEATFNTH; QIQELRKHAQ; QQDEQEVEEV; QQQQQQREKE; SGPFNLRSNE; RREQEQSHQD; EDVERLIRNQ; SERFQTRYKN; RSYNARLSEG; SERFQTRYKN; EYGDALRLPA; SERFQTRYKN; DNVIRQLDRE; YEDEQEDEEQ; VKELIFPGSA
4	β-Conglutin (*Lupinus angustifolius)*	gi|1117364351	75163/5.75	40	1338	NPDKRQAYNL; YDFYPSSTKD; LQNYRIVEFQ; NQRNFLAGSE; AIPINNPGNF; VKELIFPGSA LNGRATITIV; YEEIQRIILG; SSSGKGKPSE; DNVIRQLDRE; PIYSNKFGNF; FDQRTNRLEN; EDVERLIKNQ; QQSYFSGFSK; HRLRNPYYFS; ELVGIRNQQR; QQSYFANAQP; LLGFGINADE; EGVIVRVSKE; SRPNTLILPK; NTLEATFNTH; QIQELRKHAQ; QQDEQEVEEV; EDVERLIRNQ QQQQQQREKE; KNGQIRVLER; SGPFNLRSNE; RREQEQSHQD; EDVERLIRNQ; YEDEQEDEEQ; YEDEQEDEEQ; EYGDALRLPA; SERFQTRYKN; DNQDLRVVKL
5	β-Conglutin (*Lupinus angustifolius)*	gi|1117364351	75163/5.75	37	1223	NPDKRQAYNL; YDFYPSSTKD; LQNYRIVEFQ; FDQRTNRLEN; EHGDALRLPA; VKELIFPGSA FDQRTNRLEN; AIPINNPGNF; SGPFNLRSDE; LNGRATITIV; YEEIQRIILG; SSSRKGKPSE; QQSYFSGFSK; EGVIVRVSKE; ELVGIRNQQR; LLGFGINADE; QQSYFANAQP; HRLRNPYYFS; RREQEQSHQD; EDVERLIRNQ; EYGDALRLPA; SERFQTRYKN; RSYNARLSEG; SSSRKGKPSE; DNVIRQLDRE
6	β-Conglutin (*Lupinus angustifolius)*	gi|1117364351	75163/5.75	37	1436	EREQEQQPQY, LQNYRIVEFQ; EYGDALRLPA; QQSYFSGFSK; EGVIVRVSKE; LLGFGINADE; GRRHEEEEKG; HRLRNPYYFS; SRPNTLILPK; NTLEATFNTH; QIQELRKHAQ; QQDEQEVEEV; NQRNFLAGSE; SERFQTRYKN; DNQDLRVVKL; YEEIQRILLG; SSSRKGKPSE; RSYNARLSEG; DNVIRQLDRE; KNGQIRVLER; LNGRATITIV; AIPINNPGKF; YEDEQEDEEQ; SGPFNLRSNE; VKELIFPGSA; FDQRTNRLEN; NPDKRQAYNL; YDFYPSRTKD; RREQEQSHQD; PIYSNKFGNF; EDVERLIRNQ
7	β-Conglutin (*Lupinus angustifolius)*	gi|980951548	70658/5.75	34	899	YDFYPSSTKD; NPDKRQAYNL; LQNYRIVEFQ; EHGDALRLPA; QQSYFNGFSR; QQSYFANAQP; NTLEATFNTR; RRQRNPYYFS; SKPNTLILPK; NQRNFLAGSE; AIPINNPGNF; SGPFNLRSDE; VKQLTFPGSV; DNQNLRVVKL; YEEIQRIILG; SSSRKGKPSE; DNVIRQLDKE; QQQQQREKEG; FDQRTNRLEN; PIYSNKFGNF; EDVERLIKNQ; HSDADYILVV; LLGTTSYILNPD; EDVERLIRNQ
8	β-Conglutin (*Lupinus angustifolius)*	gi|980951548	70658/5.64	42	1056	NPDKRQAYNL; YDFYPSSTKD; LQNYRIVEFQ; EHGDALRLPA; QQSYFNGFSR; RRYNAKLSEG; GTTSYILNPD; FDQRTNRLEN; NTLEATFNTR; QQDEQEQEEV; RRQRNPYYFS; SKPNTLILPK; NQRNFLAGSE; AIPINNPGNF; SGPFNLRSDE; YERFQTLYKN; ISINASSNLR; DNQNLRVVKL; YEEIQRIILG; SSSGKGKPSE; DNVIRQLDKE; PIYSNKFGNF; QQQQQREKEG; DIFVIPAGHP; HSDADYILVV; LLGFGINADE; EDVERLIKNQ; QQSYFANAQP; VKQLTFPGSV

9	β-Conglutin *(Lupinus angustifolius)*	gi|980951548	70658/5.64	36	961	FDQRTNRLEN; NPDKRQAYNL; YDFYPSSTKD; LQNYRIVEFQ; EHGDALRLPA; QQSYFNGFSR; RRYNAKLSEG; GTTSYILNPD; NTLEATFNTR; QQDEQEQEEV; RRQRNPYYFS; SKPNTLILPK; NQRNFLAGSE; AIPINNPGNF; SGPFNLRSDE; VKQLTFPGSV; LNGRATITIV; YERFQTLYKN; DNQNLRVVKL; YEEIQRIILG; SSSGKGKPSE; DNVIRQLDKE; QQQQQREKEG; PIYSNKFGNF; LLGFGINADE; LLGFGINADE; QQSYFANAQP; EDVERLIKNQ
10	β-Conglutin *(Lupinus angustifolius)*	gi|980951548	70658/5.64	43	1055	FDQRTNRLEN; NPDKRQAYNL; YDFYPSSTKD; PIYSNKFGNF; ISINASSNLR; EDVERLIKNQ; LQNYRIVEFQ; EHGDALRLPA; QQSYFNGFSR; LLGFGINADE; QQSYFANAQP; RRQRNPYYFS; SKPNTLILPK; GTTSYILNPD; QQDEQEQEEV; NQRNFLAGSE; QQQQQREKEG; HSDADYILVV; SSSGKGKPSE; DNVIRQLDKE; LNGRATITIV; AIPINNPGNF; SGPFNLRSDE; DIFVIPAGHP; VKQLTFPGSV
11	β-Conglutin *(Lupinus angustifolius)*	gi|980951548	70658/5.64	41	1079	FDQRTNRLEN; NPDKRQAYNL; YDFYPSSTKD; SRGQEQSHQD; PIYSNKFGNF; EDVERLIKNQ; LQNYRIVEFQ; EHGDALRLPA; QGVIVRVSKE; QQSYFNGFSR; LLGFGINADE; RRQRNPYYFS; SKPNTLILPK; GTTSYILNPD; NTLEATFNTR; QIQELRKHAQ; NQRNFLAGSE; QQQQQREKEG; YERFQTLYKN; HSDADYILVV; DNQNLRVVKL; YEEIQRIILG; SSSGKGKPSE; DNVIRQLDKE; LNGRATITIV; AIPINNPGNF; NEDGQEDEEQ; SGPFNLRSDE; VKQLTFPGSV
12	β-Conglutin *(Lupinus angustifolius)*	gi|1117364351	75163/5.75	25	833	LQNYRIVEFQ; QQSYFSGFSK; LLGFGINADE; HRLRNPYYFS; SRPNTLILPK; NTLEATFNTH; NQRNFLAGSE; SERFQTRYKN; HSDADYILVV; DNQDLRVVKL; YEEIQRILLG; SSSRKGKPSE; DNVIRQLDRE; LNGRATITIV; AIPINNPGKF; SGPFNLRSNE; VKELIFPGSA; FDQRTNRLEN; NPDKRQAYNL; YDFYPSRTKD; EDVERLIRNQ
13	β-Conglutin *(Lupinus angustifolius)*	gi|980951555	69366/5.96	41	1130	RTDRLENLQN; KRQSYNLENG; YPSSSKDQQS; QEQSHQDEGV; SNKFGNFYEI; SNLRLLGFGI; IKNQQQSYFA; QGSRSDSRRQ; YRIVEFQSKP; DALRLPAGTT; YFSGFSRNTL; TPNRNPQAQD; NADENQRNFL; NAQPQQQQQR; RNPYYFSSER; NTLILPKHSD; SYILNPDDNQ; EATFNTRYEE; ELRKYAQSSS; FQTLYRNRNG; NLRVVKLAIP; IQRILLGNED; RKGKPSKSGP; LDTEVKGLTF; INNPGNFYDF; EQEDDEQRHG; FNLRSNKPIY; PGSTEDVERL; AGSEDNVIRQ; IVRVSKEQVQ
14	β-Conglutin *(Lupinus angustifolius)*	gi|980951548	70658/5.64	29	425	FDQRTNRLEN; NPDKRQAYNL; YDFYPSSTKD; EDVERLIKNQ; LQNYRIVEFQ; EHGDALRLPA; LLGFGINADE; QQSYFANAQP; RRQRNPYYFS; SKPNTLILPK; GTTSYILNPD; NTLEATFNTR; NQRNFLAGSE; QQQQQREKEG; YERFQTLYKN; DNQNLRVVKL; YEEIQRIILG; SSSGKGKPSE; DNVIRQLDKE; AIPINNPGNF; SGPFNLRSDE; VKQLTFPGSV
15	α-Conglutin (*Lupinus angustifolius*)	gi|1117484096	74150/5.12	4	72	AEVLANAFGL; RLNQVSQLKY; YNPNAGRISS; VNSLTLPILR; PVKQVFRGIP
16	α-Conglutin (*Lupinus angustifolius*)	gi|1117484096	74150/5.12	12	180	DIIAIPPGIP; AEVLANAFGL; YWTYNYGEQR; LVAINLLDTT; SLLNQLDPSP; SIISPK**SQEE; YNPNAGR**ISS; VNSLTLPILR; R**RFYIAGNPE; PVKQVFR**GIP
17	β–Conglutin (*Lupinus angustifolius*)	gi|980951555	69366/5.96	30	1031	RTDRLENLQN; YPSSSKDQQS; QEQSHQDEGV; SNKFGNFYEI; SNLRLLGFGI; IKNQQQSYFA; YRIVEFQSKP; YFSGFSRNTL; IVRVSKEQVQ; TPNRNPQAQD; GIRDQQRQQD; NADENQRNFL; NAQPQQQQQR; EATFNTRYEE; ELRKYAQSSS; EQEVRRYSAR; AGSEDNVIRQ; NLRVVKLAIP; IQRILLGNED; RKGKPSKSGP; LDTEVKGLTF; INNPGNFYDF; EQEDDEQRHG; FNLRSNKPIY; PGSTEDVERL
18	β-Conglutin (*Lupinus angustifolius*)	gi|980951555	69366/5.96	36	1098	YFSGFSRNTL; IVRVSKEQVQ; NADENQRNFL; NAQPQQQQQR; NTLILPKHSD; EATFNTRYEE; EQEDDEQRHG; AGSEDNVIRQ; IQRILLGNED; QEQSHQDEGV; SNKFGNFYEI; SNLRLLGFGI; IKNQQQSYFA; RKGKPSKSGP; LDTEVKGLTF; FNLRSNKPIY; PGSTEDVERL; ELRKYAQSSS; NSKAIFVVLV; ELRKYAQSSS; GIRDQQRQQD

19	β-Conglutin (*Lupinus angustifolius*)	gi|980951555	69366/5.96	33	1070	RTDRLENLQN; QEQSHQDEGV; SNKFGNFYEI; DEGEGNYELV; SNLRLLGFGI; IKNQQQSYFA; YRIVEFQSKP; YFSGFSRNTL; IVRVSKEQVQ; TPNRNPQAQD; GIRDQQRQQD; NADENQRNFL; NAQPQQQQQR; NTLILPKHSD; EATFNTRYEE; ELRKYAQSSS; EQEVRRYSAR; AGSEDNVIRQ; IQRILLGNED; RKGKPSKSGP; LDTEVKGLTF; EQEDDEQRHG; FNLRSNKPIY; NSKAIFVVLV; PGSTEDVERL
20	β-Conglutin (*Lupinus angustifolius*)	gi|980951555	69366/5.96	37	954	RTDRLENLQN; YPSSSKDQQS; QEQSHQDEGV; SNKFGNFYEI; SNLRLLGFGI; IKNQQQSYFA; GRREEEEEWQ; YRIVEFQSKP; DALRLPAGTT; YFSGFSRNTL; IVRVSKEQVQ; TPNRNPQAQD; NADENQRNFL; NAQPQQQQQR; PRRQRPQSRR; RNPYYFSSER; NTLILPKHSD; SYILNPDDNQ; EATFNTRYEE; ELRKYAQSSS; AGSEDNVIRQ; NLRVVKLAIP; IQRILLGNED; RKGKPSKSGP; LDTEVKGLTF; QIRVLERFDQ; INNPGNFYDF; EQEDDEQRHG; FNLRSNKPIY; PGSTEDVERL
21	β-Conglutin (*Lupinus angustifolius*)	gi|169950562	71883/5.82	32	983	NGRATITIVN; GPFNLRSNKP; QLDREVKELT; NQRTNRLENL; PDKRQVYNLE; RGQEQSHQDE; FPGSIEDVER; QNYRIIEFQS; QGDALRLPAG; QSYFSGFSKN; GVIVRVSKKQ; LIKNQQQSYF; TTSYILNPDD; IQELRKHAQS; QDEQEEEYEQ; ANAQPQQQQQ; NQNLRVAKLA; SSGEGKPSES; GEEEVRRYSD; LAGSEDNVIK; REKEGRRGRR
22	β-Conglutin (*Lupinus angustifolius*)	gi|169950562	71883/5.82	29	1033	GPFNLRSNKP; QLDREVKELT; NQRTNRLENL; DFYPSTTKDQ; RGQEQSHQDE; FPGSIEDVER; QNYRIIEFQS; QGDALRLPAG; QSYFSGFSKN; GVIVRVSKKQ; LVGIRDQQRQ; SSNLRLLGFG; LIKNQQQSYF; TTSYILNPDD; TLEATFNTRY; IQELRKHAQS; QDEQEEEYEQ; INANENQRNF; ANAQPQQQQQ; NQNLRVAKLA; SSGEGKPSES; GEEEVRRYSD; LAGSEDNVIK; REKEGRRGRR
23	β-Conglutin (*Lupinus angustifolius*)	gi|980951555	69366/5.96	24	480	RTDRLENLQN; KRQSYNLENG; IKNQQQSYFA; QGSRSDSRRQ; YRIVEFQSKP; DALRLPAGTT; YFSGFSRNTL; NADENQRNFL; NAQPQQQQQR; AGSEDNVIRQ; NLRVVKLAIP; IQRILLGNED; SNLRLLGFGI; PGSTEDVERL; LDTEVKGLTF
24	α-Conglutin (*Lupinus angustifolius*)	gi|328684559	57758/5.26	33	533	TIETWNPNND; QGQQQEGGNE; VKEGLKVISP; RLKTLTSLDF; QLRCAGVALS; YEEPQEQEQG; GGNVLSGFND; PTLRPRQGRE; SSIRALPLDV; RCTIQRNGLR; QGPRPQDRHQ; DNQLDQIPRR; EFLEEAFSVD; EFLEEAFSVD; VAHAFNLDRD; CQFQRLNALE; RPFYTNAPQE; KVEHFREGDI; FYLSGNQEQE; REIVRNIKGK; QARQLKNNNP; PDNSVKSEAG; IYIQQGRGIF; FLQYQQKEGG
25	β-Conglutin (*Lupinus angustifolius*)	gi|980951555	69366/5.96	37	996	YPSSSKDQQS; SNLRLLGFGI; QEQSHQDEGV; SNKFGNFYEI; IKNQQQSYFA; YRIVEFQSKP; DALRLPAGTT; YFSGFSRNTL; TPNRNPQAQD; IVRVSKEQVQ; NADENQRNFL; NAQPQQQQQR; RNPYYFSSER; SYILNPDDNQ; EATFNTRYEE; AGSEDNVIRQ; FQTLYRNRNG; NLRVVKLAIP; IQRILLGNED; RKGKPSKSGP; LSEGDIFVIP; LDTEVKGLTF; QIRVLERFDQ; INNPGNFYDF; EQEDDEQRHG; FNLRSNKPIY; AGHPISINAS; PGSTEDVERL; RTDRLENLQN
26	β-Conglutin (*Lupinus angustifolius*)	gi|980951550	68139/5.71	23	763	QGSRSDSRRQ; YRIVEFQSKP; DALRLPAGTT; YFSGFSKNTL; NADENQRNFL; RNPYYFSSER; FQTLYRNRNG; NTLILPKHSD; EATFNTRYEE; AGSEDNVIRQ; NLRVVKLAIP; QIRVLERFDQ; INNPGNFYDF; RTNRLENLQN; KRQSYNLENG; YPSSSKDQQS
27	β-Conglutin (*Lupinus angustifolius*)	gi|980951555	68139/5.71	23	678	QGSRSDSRRQ; YRIVEFQSKP; DALRLPAGTT; YFSGFSKNTL; NADENQRNFL; RNPYYFSSER; NTLILPKHSD; SYILNPDDNQ; EATFNTRYEE; AGSEDNVIRQ; FQTLYRNRNG; NLRVVKLAIP; QIRVLERFDQ; INNPGNFYDF; RTNRLENLQN; KRQSYNLENG; YPSSSKDQQS
28	β-Conglutin (*Lupinus angustifolius*)	gi|980951550	69366/5.96	28	764	RTDRLENLQN; KRQSYNLENG; YPSSSKDQQS; IKNQQQSYFA; GRREEEEEWQ; IQRILLGNED; YRIVEFQSKP; DALRLPAGTT; YFSGFSKNTL; IVRVSKEQVQ; NADENQRNFL; NAQPQQQQQR; PRRQRPQSRR; RNPYYFSSER; NTLILPKHSD; ELRKYAQSSS; AGSEDNVIRQ; FQTLYRNRNG; NLRVVKLAIP; QGSRSDSRRQ; INNPGNFYDF
29	β-Conglutin (*Lupinus angustifolius*)	gi|1117364351	75163/5.75	21	615	NPDKRQAYNL; YDFYPSSTKD; LQNYRIVEFQ; GTTSYILNPD; AIPINNPGNF; LNGRATITIV; YEEIQRIILG; FDQRTNRLEN; QQSYFSGFSK; HRLRNPYYFS; SRPNTLILPK; NTLEATFNTH; KNGQIRVLER; EYGDALRLPA; SERFQTRYKN; DNQDLRVVKL

30	β-Conglutin (*Lupinus angustifolius*)	gi|1117364351	75163/5.75	20	577	QQSYFSGFSK; EGVIVRVSKE; YEITPDRNPQ; LLGFGINADE; QIQELRKHAQ; VQDLDISLIF; NQRNFLAGSE; HSDADYILVV; SSSRKGKPSE; TEISEGALLL; SGPFNLRSNE; RREQEQSHQD; PHYNSKAIFV; PIYSNKFGNF
31	α-Conglutin (*Lupinus angustifolius*)	gi|1117385770	67340/5.31	8	196	NIDEDTVHK**L; QNPNERIK**QI; IRVEEGLGVI; **QEGK**NNILSG; SPK**WQEQEEE;** EPDNR**IESEG;** FDPQFLSQAL
32	α-Conglutin (*Lupinus angustifolius*)	gi|1117523166	57758/5.26	35	755	TIETWNPNND; GLIFPGCRET; QGQQQEGGNE; VKEGLKVISP; EEWSHQVRRV; QLRCAGVALS; YEEPQEQEQG; GGNVLSGFND; PTLRPRQGRE; QGPRPQDRHQ; DNQLDQIPRR; EFLEEAFSVD; CQFQRLNALE; RPFYTNAPQE; KVEHFREGDI; FYLSGNQEQE; REIVRNIKGK; ERRGDRRRHR; PDNSVKSEAG; IYIQQGRGIF; FLQYQQKEGG; NDDREGSIVE; PHHHEEEEEE
33	β-Conglutin (*Lupinus angustifolius*)	gi|980951555	69366/5.96	41	1112	RTDRLENLQN; YPSSSKDQQS; QEQSHQDEGV; SNKFGNFYEI; DEGEGNYELV; SNLRLLGFGI; IKNQQQSYFA; QGSRSDSRRQ; YRIVEFQSKP; DALRLPAGTT; YFSGFSRNTL; IVRVSKEQVQ; TPNRNPQAQD; GIRDQQRQQD; NADENQRNFL; NAQPQQQQQR; RNPYYFSSER; SYILNPDDNQ; EATFNTRYEE; ELRKYAQSSS; AGSEDNVIRQ; FQTLYRNRNG; NLRVVKLAIP; RKGKPSKSGP; LSEGDIFVIP; LDTEVKGLTF; QIRVLERFDQ; INNPGNFYDF; EQEDDEQRHG; FNLRSNKPIY; NSKAIFVVLV; AGHPISINAS; PGSTEDVERL
34	β-Conglutin (*Lupinus angustifolius*)	gi|980951555	69366/5.96	31	567	RTDRLENLQN; KRQSYNLENG; YPSSSKDQQS; SNLRLLGFGI; YRIVEFQSKP; DALRLPAGTT; YFSGFSRNTL; NADENQRNFL; RNPYYFSSER; NTLILPKHSD; SYILNPDDNQ; EATFNTRYEE; AGSEDNVIRQ; FQTLYRNRNG; NLRVVKLAIP; IQRILLGNED; LSEGDIFVIP; LDTEVKGLTF; QIRVLERFDQ; INNPGNFYDF; AGHPISINAS
35	β-Conglutin (*Lupinus angustifolius*)	gi|1117364351	75163/5.75	41	1423	LQNYRIVEFQ; EYGDALRLPA; QQSYFSGFSK; EGVIVRVSKE; LLGFGINADE; QQSYFANAQP; HRLRNPYYFS; GTTSYILNPD; NTLEATFNTH; QIQELRKHAQ; QQDEQEVEEV; NQRNFLAGSE; QQQQQQREKE; SERFQTRYKN; HSDADYILVV; DNQDLRVVKL; YEEIQRILLG; SSSRKGKPSE; RSYNARLSEG; DNVIRQLDRE; KNGQIRVLER; LNGRATITIV; AIPINNPGKF; YEDEQEDEEQ; SGPFNLRSNE; VKELIFPGSA; FDQRTNRLEN; NPDKRQAYNL; YDFYPSRTKD; RREQEQSHQD; EDVERLIRNQ
36	β-Conglutin (*Lupinus angustifolius*)	gi|1117364351	75163/5.75	45	1601	EQEQGSSSES; LQNYRIVEFQ; EYGDALRLPA; QQSYFSGFSK; EGVIVRVSKE; LLGFGINADE; QQSYFANAQP; YEITPDRNPQ; GRRHEEEEKG; HRLRNPYYFS; SRPNTLILPK; GTTSYILNPD; NTLEATFNTH; QIQELRKHAQ; QQDEQEVEEV; NQRNFLAGSE; QQQQQQREKE; KNGQIRVLER; LNGRATITIV; AIPINNPGKF; YEDEQEDEEQ; SGPFNLRSNE; VKELIFPGSA; FDQRTNRLEN; NPDKRQAYNL; YDFYPSRTKD; RREQEQSHQD; PIYSNKFGNF; EDVERLIRNQ
37	β-Conglutin (*Lupinus angustifolius*)	gi|1117364351	75163/5.75	43	1412	EQEQGSSSES; LQNYRIVEFQ; EYGDALRLPA; QQSYFSGFSK; EGVIVRVSKE; LLGFGINADE; QQSYFANAQP; HRLRNPYYFS; SRPNTLILPK; GTTSYILNPD; NTLEATFNTH; QIQELR**KHAQ;** QQDEQEVEEV; NQRNFLAGSE; QQQQQQR**EKE;** SER**FQTRYKN;** DNQDLR**VVK**L; YEEIQRILLG; **SSSRK**GKPSE; RSYNAR**LSEG;** DNVIRQLDRE; **KNGQIR**VLER; **LNGR**ATITIV; AIPINNPGKF; YEDEQEDEEQ; SGPFNLR**SNE;** VKELIFPGSA; FDQRTNRLEN; NPDKRQAYNL; YDFYPSRTKD; RREQEQSHQD; EDVER**LIR**NQ
38	β-Conglutin (*Lupinus angustifolius*)	gi|1117364351	75163/5.75	40	1194	EQEQGSSSES; LQNYRIVEFQ; EYGDALRLPA; QQSYFSGFSK; EGVIVRVSKE; LLGFGINADE; QQSYFANAQP; HRLRNPYYFS; SRPNTLILPK; GTTSYILNPD; QIQELRKHAQ; QQDEQEVEEV; NQRNFLAGSE; QQQQQQREKE; SERFQTRYKN; DNQDLRVVKL; YEEIQRILLG; SSSRKGKPSE; RSYNARLSEG; DNVIRQLDRE; KNGQIRVLER; LNGRATITIV; AIPINNPGKF; YEDEQEDEEQ; SGPFNLRSNE; VKELIFPGSA; FDQRTNRLEN; NPDKRQAYNL; YDFYPSRTKD; RREQEQSHQD; EDVERLIRNQ

39	β-Conglutin (*Lupinus angustifolius*)	gi|980951555	69366/5.96	26	611	YPSSSKDQQS; **SNK**FGNFYEI; SNLRLLGFGI; **YR**IVEFQSKP; YFSGFSRNTL; TPNR**NPQAQD;** NADENQRNFL; **R**NPYYFSSER; NTLILPK**HSD;** EATFNTRYEE; AGSEDNVIR**Q;** FQTLYR**NRNG; NLRVVK**LAIP; IQR**ILLGNED; RKGKPSK**SGP; LSEGDIFVIP; **LDTEVK**GLTF; INNPGNFYDF; FNLR**SNKPIY;** AGHPISINAS; PGSTEDVER**L**
40	β-Conglutin (*Lupinus angustifolius*)	gi|980951548	70658/5.64	29	677	YDFYPSSTKD; ISINASSNLR; HSDADYILVV**;** QQSYFNGFSR; LLGFGINADE; SKPNTLILPK; NTLEATFNTR; NQRNFLAGSE; YERFQTLYK**N;** LNGRATITIV; AIPINNPGNF; DIFVIPAGHP
41	β-Conglutin (*Lupinus angustifolius*)	gi|980951548	70658/5.64	35	956	NPDKRQAYNL; YDFYPSSTKD; LQNYRIVEFQ; EHGDALRLPA; QQSYFNGFSR; LLGFGINADE; SKPNTLILPK; GTTSYILNPD; NTLEATFNTR; NQRNFLAGSE; HSDADYILVV; LNGRATITIV; SGPFNLRSDE
42	β-Conglutin (*Lupinus angustifolius*)	gi|980951548	70658/5.64	36	936	NPDKRQAYNL; YDFYPSSTKD; LQNYRIVEFQ; EHGDALRLPA; QQSYFNGFSR; LLGFGINADE; SKPNTLILPK; NTLEATFNTR; NQRNFLAGSE; HSDADYILVV; LNGRATITIV; AIPINNPGNF
43	β-Conglutin (*Lupinus angustifolius*)	gi|1117308442	70658/5.64	40	955	NPDKRQAYNL; YDFYPSSTKD; EDVERLIKNQ; EHGDALRLPA; QQSYFNGFSR; LLGFGINADE; QQSYFANAQP; GTTSYILNPD; NTLEATFNTR; NQRNFLAGSE; HSDADYILVV; LNGRATITIV; AIPINNPGNF; DIFVIPAGHP
45	α-Conglutin (*Lupinus angustifolius*)	gi|1117484096	74150/5.12	8	171	DIIAIPPGIP; YWTYNYGEQR; LVAINLLDTT; KQIVRVKRGL; SLLNQLDPSP; SIISPKSQEE
46	β-Conglutin (*Lupinus angustifolius*)	gi|1117364351	75163/5.75	4	78	LQNYRIVEFQ; LNGRATITIV; FDQRTNRLEN; NPDKRQAYNL; YDFYPSRTKD
47	β-Conglutin (*Lupinus angustifolius*)	gi|980951550	68139/5.71	8	187	YR**IVEFQSKP;** NADENQR**NFL;** FQTLYR**NRNG; NLRVVK**LAIP; INNPGNFYDF; **R**TNRLENLQN; YPSSSK**DQQS; SNLR**LLGFGI
48	α-Conglutin (*Lupinus angustifolius*)	gi|1117484096	74150/5.12	7	123	DIIAIPPGIP; YWTYNYGEQR; LVAINLLDTT; **KQIVRVKR**GL; SLLNQLDPSP; SIISPK**SQEE; HQKIQYFR**EG; R**RFYIAGNPE**
49	α-Conglutin (*Lupinus angustifolius*)	gi|1117523166	57758/5.26	10	127	TIETWNPNND; **VKEGLK**VISP; RPFYTNAPQE**;** QLR**CAGVALS;** PTLRPR**QGRE; CQFQR**LNALE; PDNSVKSEAG
50	β-Conglutin (*Lupinus angustifolius*)	gi|980951555	69366/5.96	40	967	R**TDRLENLQN;** YPSSSK**DQQS;** QEQSHQDEGV; SNKFGNFYEI; SNLRLLGFGI; IKNQQQSYFA; **QGSRSDSRR**Q; **YR**IVEFQSKP; **DALR**LPAGTT; **YFSGFSR**NTL; IVRVSKEQVQ; TPNR**NPQAQD;** NADENQRNFL; NAQPQQQQQR; RNPYYFSSER; NTLILPK**HSD;** SYILNPDDNQ; EATFNTRYEE; ELR**KYAQSSS;** AGSEDNVIRQ; FQTLYR**NRNG;** NLR**VVK**LAIP; IQR**ILLGNED; RKGKPSK**SGP; LSEGDIFVIP; LDTEVKGLTF; **QIR**VLERFDQ; INNPGNFYDF; **EQEDDEQR**HG; FNLRSNKPIY; AGHPISINAS; PGSTEDVERL
51	β-Conglutin (*Lupinus angustifolius*)	gi|980951555	69366/5.96	30	478	KRQSYNLENG; YPSSSKDQQS; SNLRLLGFGI; QGSRSDSRRQ; YRIVEFQSKP; DALRLPAGTT; YFSGFSRNTL; IVRVSKEQVQ; NADENQRNFL; RNPYYFSSER; NTLILPKHSD; SYILNPDDNQ; EATFNTRYEE; ELRKYAQSSS; AGSEDNVIRQ; FQTLYRNRNG; NLRVVKLAIP; IQRILLGNED; RKGKPSKSGP; LSEGDIFVIP; LDTEVKGLTF; INNPGNFYDF; FNLRSNKPIY; AGHPISINAS; PGSTEDVERL
52	β-Conglutin (*Lupinus angustifolius*)	gi|980951555	69366/5.96	22	743	YPSSSKDQQS; QEQSHQDEGV; SNKFGNFYEI; SNLRLLGFGI; IKNQQQSYFA; IVRVSKEQVQ; TPNRNPQAQD; NADENQRNFL; NAQPQQQQQR; ELRKYAQSSS; AGSEDNVIRQ; NLRVVKLAIP; RKGKPSKSGP; LDTEVKGLTF; INNPGNFYDF; EQEDDEQRHG; FNLRSNKPIY; PGSTEDVERL
53	β-Conglutin (*Lupinus angustifolius*)	gi|980951555	69366/5.96	25	848	YPSSSKDQQS; QEQSHQDEGV; SNKFGNFYEI; SNLRLLGFGI; IKNQQQSYFA; YFSGFSRNTL; EATFNTRYEE; IVRVSKEQVQ; TPNRNPQAQD; NADENQRNFL; NAQPQQQQQR; ELRKYAQSSS; AGSEDNVIRQ; NLRVVKLAIP; RKGKPSKSGP; LDTEVKGLTF; INNPGNFYDF; EQEDDEQRHG; FNLRSNKPIY; PGSTEDVERL

54	β-Conglutin (*Lupinus angustifolius*)	gi|980951555	69366/5.96	23	866	YPSSSKDQQS; QEQSHQDEGV; SNKFGNFYEI; SNLRLLGFGI; IKNQQQSYFA; IVRVSKEQVQ; TPNRNPQAQD; NADENQRNFL; NAQPQQQQQR; ELRKYAQSSS; AGSEDNVIRQ; NLRVVKLAIP; RKGKPSKSGP; LDTEVKGLTF; INNPGNFYDF; EQEDDEQRHG; FNLRSNKPIY; PGSTEDVERL
55	β-Conglutin (*Lupinus angustifolius*)	gi|980951550	68139/5.71	41	832	YRIVEFQSKP; DALRLPAGTT; YFSGFSKNTL; TPDRNPQAQD; GIR**DQQRQQD;** NADENQRNFL; NAQPQQQQQR; NTLILPK**HSD;** SYILNPDDNQ; EATFNTR**YEE;** AGSEDNVIR**Q;** FQTLYR**NRNG;** NLR**VVK**LAIP; **R**KGKPYESGP; NEGALLLPHY; **LDREVK**GLIF; **QIR**VLERFDQ; INNPGNFYDF; FNLR**SNKPIY;** NSKAIFVVVV; PGSAEDVER**L;** RTNRLENLQN; **KR**QSYNLENG; YPSSSKDQQS; **IK**NQQQSYFA**SNK**FGNFYEI; DEGEGNYELV; **SNLR**LLGFGI; **IK**NQQQSYFA
56	β-Conglutin (*Lupinus angustifolius*)	gi|980951548	70658/5.64	34	881	FDQRTNRLEN; NPDKRQAYNL; YDFYPSSTKD; EDVERLIKNQ; LQNYRIVEFQ; EHGDALRLPA; QQSYFNGFSR; LLGFGINADE; QQSYFANAQP; RRQRNPYYFS; GTTSYILNPD; NTLEATFNTR; NQRNFLAGSE; QQQQQREKEG; YERFQTLYKN; HSDADYILVV; SGPFNLRSDE; DNQNLRVVKL; YEEIQRIILG; SSSGKGKPSE; RNGQIRVLER; LNGRATITIV; SKPNTLILPK; DNVIRQLDKE
57	β-Conglutin (*Lupinus angustifolius*)	gi|980951550	68139/5.71	30	895	EEEEEWQPRR; YRIVEFQSKP; DALRLPAGTT; IVRVSKEQVQ; NADENQRNFL; NAQPQQQQQR; QRPQSRREER; RNPYYFSSER; EATFNTRYEE; ELRKYAQSSS; AGSEDNVIRQ; FQTLYRNRNG; NLRVVKLAIP; RKGKPYESGP; LDREVKGLIF; QIRVLERFDQ; INNPGNFYDF; FNLRSNKPIY; PGSAEDVERL; RTNRLENLQN; KRQSYNLENG; YPSSSKDQQS, SNLRLLGFGI; IKNQQQSYFA
58	β-Conglutin (*Lupinus angustifolius*)	gi|1117364351	75163/5.75	22	623	LQNYRIVEFQ; EGVIVRVSKE; YEITPDRNPQ; LLGFGINADE; HRLRNPYYFS; QIQELRKHAQ; QQDEQEVEEV; NQRNFLAGSE; HSDADYILVV; SSSRKGKPSE; DNVIRQLDRE; LNGRATITIV; SGPFNLRSNE; DILVIPAGHP; VKELIFPGSA; LSINASSNLR; EDVERLIRNQ
59	β-Conglutin (*Lupinus angustifolius*)	gi|980951555	69366/5.96	42	901	RTDRLENLQN; **KR**QSYNLENG; YPSSSKDQQS; **SNK**FGNFYEI; SNLRLLGFGI; **IK**NQQQSYFA; **GR**REEEEEWQ; YRIVEFQSKP; DALRLPAGTT; YFSGFSRNTL; **IVR**VSKEQVQ; TPNR**NPQAQD;** INNPGNFYDF; NTLILPK**HSD;** SYILNPDDNQ; EATFNTRYEE; ELR**KYAQSSS;** AGSEDNVIRQ; FQTLYR**NRNG;** NLR**VVK**LAIP; IQR**ILLGNED;** **RKGKPSK**SGP; LSEGDIFVIP; LDTEVKGLTF; **QIR**VLERFDQ; FNLR**SNKPIY;** AGHPISINAS; PGSTEDVER**L;** NADENQRNFL; **R**NPYYFSSER NAQPQQQQQR; PR**RQRPQSRR**
60	β-Conglutin (*Lupinus angustifolius*)	gi|980951555	69366/5.96	38	873	RTDRLENLQN; KRQSYNLENG; YPSSSKDQQS; SNKFGNFYEI; GRREEEEEWQ; YRIVEFQSKP; DALRLPAGTT; YFSGFSRNTL; TPNRNPQAQD; NADENQRNFL; PRRQRPQSRR; RNPYYFSSER; SYILNPDDNQ; AGSEDNVIRQ; FQTLYRNRNG; NLRVVKLAIP; IQRILLGNED; RKGKPSKSGP; LSEGDIFVIP; LDTEVKGLTF; INNPGNFYDF; EQEDDEQRHG; FNLRSNKPIY; AGHPISINAS; PGSTEDVERL
61	β-Conglutin (*Lupinus angustifolius*)	gi|1117308442	70658/5.64	39	866	FDQRTNRLEN; NPDKRQAYNL; YDFYPSSTK**D;** ISINASSNLR; **GR**REEEEEWQ; LQNYRIVEFQ; EHGDALRLPA; SKPNTLILPK; NTLEATFNTR**;** GTTSYILNPD; NQRNFLAGSE; HSDADYILVV; LNGRATITIV; AIPINNPGNF; DIFVIPAGHP
62	β-Conglutin (*Lupinus angustifolius*)	gi|1117364351	75163/5.75	11	339	QQSYFSGFSK; LLGFGINADE; HRLRNPYYFS; NQRNFLAGSE; SERFQTRYKN; SSSRKGKPSE; LNGRATITIV; SGPFNLRSNE; NPDKRQAYNL; YDFYPSRTKD
63	β-Conglutin (*Lupinus angustifolius*)	gi|980951548	70658/5.64	23	582	NPDKRQAYNL; FDQRTNRLEN; YDFYPSSTKD; SRGQEQSHQD; PIYSNKFGNF; EDVERLIKNQ; LQNYRIVEFQ; LLGFGINADE; YERFQTLYKN; LNGRATITIV; NEDGQEDEEQ; SGPFNLRSDE
64	β-Conglutin (*Lupinus angustifolius*)	gi|1117364351	75163/5.75	11	200	LQNYRIVEFQ; LLGFGINADE; HSDADYILVV; LNGRATITIV; NPDKRQAYNL; NQRNFLAGSE; FDQRTNRLEN
65	β-Conglutin (*Lupinus angustifolius*)	gi|980951548	70658/5.64	23	582	FDQRTNR**LEN;** **NPDKR**QAYNL; **YDFYPSSTK**D; **PIYSNK**FGNF; **LLGFGINADE;** **SKPNTLILPK;** **QIQELR**KHAQ; **YERFQTLYK**N; SSSGK**GKPSE; AIPINNPGNF; SGPFNLRSDE; NEDGQEDEEQ**

66	β-Conglutin (*Lupinus angustifolius*)	gi|169950562	71883/5.82	10	233	NQR**TNRLENL; FPGSIEDVER,** SSNLR**LLGFG; TLEATFNTR**Y; **INANENQRNF; LAGSEDNVIK**
67	hypothetical protein Tanjilg *(Lupinus angustifolius)*	gi|1117575924	41222/5.81	36	533	KVIKVPEGFD; VRGYPFSLRE; PKGTLFPMCG; TDHLGLGVKT; KDSTSVQQCY; AKSSKAANGT; LLKDELDIVI; YELYNRNDIN; MNLAFDRELI; GLPYIWHSKA; IELSKQVKEK; PTIRNLDFLE; GPAMYFGLMG; SNPFVNLKKE; LSPIDPYFTK; DGQPIGRYDD; YKGIFWQEEI; LADAMVTWIE; YDPYREGADF; TRYVDAVLTI; IPFFQNATLS; MWAGWCVKVI; AWDELNTPSE
68	hypothetical protein Tanjilg *(Lupinus angustifolius)*	gi|1117298147	71243/5.27	28	798	RLIGDAAKNQ; GDKPMIVVNY; AYFNDSQRQA; TITRARFEEM; VQQLLQDFFN; VAMNPHNTVF; KGEEKTFSAE; TKDAGAISGL; NMDLFRKCME; GKELCKSINP; GVWQNDRVEI; DAKRLIGRRV; EISSMVLTKM; NVLRIINEPT; IFEVKATAGD, IPNDQGNRTT; REIAEAFLGQ; AAAIAYGLDK; THLGGEDFDN; IDKSQIHEVV; PSYVAFTDTE; LWPFKVIPGA;<br>PVKNAVVTVP; KASRKGEQNV; RMVNHFVSEF; LVGGSTRIPK; LLLKLNPLVS; DGGTEVVEAK
69	α-Conglutin (*Lupinus angustifolius*)	gi|980951555	69366/5.96	22	353	R**TDRLENLQN;** KR**QSYNLENG; DALRLPAGTT; SYILNPDDNQ; EATFNTRYEE; AGSEDNVIR**Q; **LSEGDIFVIP; AGHPISINAS; PGSTEDVER**L
70	α-Conglutin (*Lupinus angustifolius*)	gi|1117523166	57758/5.26	5	64	PQIAALAGLT; **RLK**TLTSLDF; PILR**WLGLAA;** SSIR**ALPLDV; SYVAFK**TNDI
71	β-Conglutin (*Lupinus angustifolius*)	gi|980951550	68139/5.71	6	121	YFSGFSKNTL; EATFNTR**YEE;** FQTLYR**NRNG; R**TNRLENLQN; **YPSSSK**DQQS; YR**IVEFQSKP**
72	β-Conglutin (*Lupinus angustifolius*)	gi|980951548	70658/5.64	25	487	FDQRTNRLEN; NPDKRQAYNL; YDFYPSSTKD; ISINASSNLR; EDVERLIKNQ; GRREEEEEWQ; LQNYRIVEFQ; EHGDALRLPA; QGVIVRVSKE; LLGFGINADE; PRRQRPQSRR; GTTSYILNPD; QIQELRKHAQ; NQRNFLAGSE; DNQNLRVVKL; RRYNAKLSEG; DNVIRQLDKE; LNGRATITIV; AIPINNPGNF; DIFVIPAGHP; VKQLTFPGSV
73	α-Conglutin (*Lupinus angustifolius*)	gi|1117385770	67340/5.31	19	326	GVTETWNSNK; YPETQQQRQQ; NIDEDTVHKL; PELRCAGVAF; QNPNERIKQI; RQERRRGQRS; IRVEEGLGVI; ECQLDRLNAL; QSQEQEDSHQ; QEGKNNILSG; SPKWQEQEEE; EPDNRIESEG; KIRHFREGDI; FYLAGNPEEE; FDPQFLSQAL; EEEKEEPRQR
74	β-Conglutin (*Lupinus angustifolius*)	gi|980951555	69366/5.96	12	248	RTDRLENLQN, YPSSSKDQQS, SNLRLLGFGI, NADENQRNFL, EATFNTRYEE, INNPGNFYDF, FQTLYRNRNG
75	β-Conglutin (*Lupinus angustifolius*)	gi|980951550	68139/5.71	8	175	YFSGFSKNTL; NADENQRNFL; RNPYYFSSER; FQTLYRNRNG; RTNRLENLQN; SNLRLLGFGI
76	β-Conglutin (*Lupinus angustifolius*)	gi|980951550	70658/5.64	3	94	FDQRTNRLEN; LQNYRIVEFQ; LLGFGINADE; NQRNFLAGSE
77	β-Conglutin (*Lupinus angustifolius*)	gi|149208403	54267/6.27	8	78	QNYRIIEFQS; RQRNPYHFSS; KPNTLILPKH; SDADFILVVL; NGRATITIVN
78	β-Conglutin (*Lupinus angustifolius*)	gi|980951555	69366/5.96	6	99	EATFNTRYEE; LSEGDIFVIP; AGHPISINAS; FNLRSNKPIY; AGHPISINAS; YFSGFSRNTL
79	β-Conglutin (*Lupinus angustifolius*)	gi|980951555	69366/5.96	17	481	YPSSSKDQQS; QEQSHQDEGV; SNKFGNFYEI; SNLRLLGFGI; IVRVSKEQVQ; TPNRNPQAQD; NADENQRNFL; AGSEDNVIRQ; INNPGNFYDF; LDTEVKGLTF
80	β-Conglutin (*Lupinus angustifolius*)	gi|980951550	68139/5.71	15	428	IVRVSKEQVQ; NADENQRNFL; AGSEDNVIRQ; RKGKPYESGP; FNLRSNKPIY; PGSAEDVERL; SNKFGNFYEI; SNLRLLGFGI
81	hypothetical protein Tanjilg *(Lupinus angustifolius)*	gi|1102723386	38596/6.47	36	639	ITTDYMTYMF; FIGDNRSSIF; WRGGRAASFN; DAKAGIALNE; IIPSSTGAAK; TLLFGEKSVA; AKKVIISAPS; DRFGIVEGLM; VELVAVNDPF; TTVHSITATQ; GKLTGMAFRV; YTEDDVVST

82	β-Conglutin (*Lupinus angustifolius*)	gi|980951550	75163/5.75	12	400	QQSYFSGFSK; LLGFGINADE; HRLRNPYYFS; NQRNFLAGSE; LNGRATITIV; NPDKRQAYNL
83	β-Conglutin (*Lupinus angustifolius*)	gi|980951555	69366/5.96	16	296	YPSSSKDQQS; YRIVEFQSKP; NADENQRNFL; IQRILLGNED; INNPGNFYDF; EQEDDEQRHG; FNLRSNKPIY
84	β-Conglutin (*Lupinus angustifolius*)	gi|980951548	68139/5.71	17	298	YRIVEFQSKP; YFSGFSKNTL; NADENQRNFL; EATFNTRYEE; LSEGDI; FVIP; INNPGNFYDF; AGHPISINAS; YPSSSKDQQS; SNLRLLGFGI
85	β-Conglutin (*Lupinus angustifolius*)	gi|980951548	70658/5.64	26	615	NPDKRQAYNL; YDFYPSSTKD; ISINASSNLR; LQNYRIVEFQ; EHGDALRLPA; QQSYFNGFSR; NQRNFLAGSE; DIFVIPAGHP; AIPINNPGNF
86	β-Conglutin (*Lupinus angustifolius*)	gi|980951548	70658/5.64	34	882	NPDKRQAYNL; YDFYPSSTKD; EDVERLIKNQ; LQNYRIVEFQ; EHGDALRLPA; QQSYFNGFSR; LLGFGINADE; GTTSYILNPD; NTLEATFNTR; NQRNFLAGSE; HSDADYILVV; NEDGQEDEEQ
87	β-Conglutin (*Lupinus angustifolius*)	gi|980951548	70658/5.64	28	701	FDQRTNRLEN; NPDKRQAYNL; YDFYPSSTKD; QQDEQEQEEV; NQRNFLAGSE; YERFQTLYKN; HSDADYILVV; SSSGKGKPSE; LNGRATITIV
88	β-Conglutin (*Lupinus angustifolius*)	gi|980951548	66974/5.44	25	646	YDFYPSSTKD; PIYSNKFGNF; EDVERLIKNQ; QQSYFNGFSR; LLGFGINADE; YERFQTLYKN<br>SKPNTLILPK; QQDEQEQEEV; NQRNFLAGSE; HSDADYILVV; DNVIRQLDKE; VKQLTFPGSV
89	α-Conglutin (*Lupinus angustifolius*)	gi|1117523166	57758/5.26	5	60	RLKTLTSLDF; PQIAALAGLT; PILRWLGLAA; SSIRALPLDV; SYVAFKTNDI
90	hypothetical protein Tanjilg *(Lupinus angustifolius)*	gi|1117433037	46631/6.40	24	803	DVASGAGQYS; TGEAYEKTSE; KAKDGYDAAK; ESWTGWAKEK; EKTKDYAGSA; AEKAREMKDA; DTIASNLEAA; LSEGLGLKND; ENKDSTTKKA; AAQKTKEKVQ; TGTSGSAKDK; SNEELNWAKE
91	hypothetical protein Tanjilg *(Lupinus angustifolius)*	gi|1102695803	46631/6.40	26	693	ETAKEGKEAT; DVASGAGQYS; TGEAYEKTSE; KAKDGYDAAK; ESWTGWAKEK; EKTKDYAGSA; AEKAREMKDA; SINEAKERTY; DTIASNLEAA; LSEGLGLKND; AEKTKDYAGS; ATEKASDIAN; KQKSQEVKDK; AAKEKIKNVA; SEEANERQRE; LGGQRRDAEL; AAQKTKEKVQ; TGTSGSAKDK; SNEELNWAKE
92	hypothetical protein Tanjilg *(Lupinus angustifolius)*	gi|1117433037	46631/6.40	25	738	KTKEYAGDAA; ARESAGDAAQ; DVASGAGQYS; TGEAYEKTSE; KAKDGYDAAK; ESWTGWAKEK; EKTKDYAGSA; KTKDYTGSAA; AEKAREMKDA; SINEAKERTY; DTIASNLEAA; LSEGLGLKND; AEKTKDYAGS; ATEKASDIAN; KQKSQEVKDK; AAKEKIKNVA; AAKEKIKNVA; TNDYAGSAAG; AAQKTKEKVQ; SNEELNWAKE
93	α-Conglutin (*Lupinus angustifolius*)	gi|1117523166	57758/5.26	9	98	QGQQQEGGNE; GGNVLSGFND; EFLEEAFSVD; CQFQRLNALE; REIVRNIKGK; PDNSVKSEAG; FLQYQQKEGG
94	β-Conglutin (*Lupinus angustifolius*)	gi|980951550	68139/5.71	5	84	YFSGFSKNTL; NLRVVKLAIP; INNPGNFYDF; YPSSSKDQQS
95	β-Conglutin (*Lupinus angustifolius*)	gi|980951550	68139/5.71	4	68	NLRVVKLAIP; FQTLYRNRNG; INNPGNFYDF; YPSSSKDQQS
96	β-Conglutin (*Lupinus angustifolius*)	gi|980951550	68139/5.71	6	122	YFSGFSKNTL; RNPYYFSSER; NLRVVKLAIP; INNPGNFYDF; YPSSSKDQQ<br>S
97	β-Conglutin (*Lupinus angustifolius*)	gi|980951555	69366/5.96	17	646	QEQSHQDEGV; SNKFGNFYEI; SNLRLLGFGI; IVRVSKEQVQ; NADENQRNFL; AGSEDNVIRQ; RKGKPSKSGP; LDTEVKGLTF; FNLRSNKPIY; PGSTEDVERL
98	β-Conglutin (*Lupinus angustifolius*)	gi|980951555	69366/5.96	23	717	NADENQRNFL; IVRVSKEQVQ; NAQPQQQQQR; ELRKYAQSSS; EQEVRRYSAR; AGSEDNVIRQ; LDTEVKGLTF; PGSTEDVERL; FNLRSNKPIY
99	β-Conglutin (*Lupinus angustifolius*)	gi|980951555	69366/5.96	25	644	QEQSHQDEGV; SNKFGNFYEI; IVRVSKEQVQ; NADENQRNFL; NAQPQQQQQR; AGSEDNVIRQ; LDTEVKGLTF; INNPGNFYDF; FNLRSNKPIY

100	β-Conglutin (*Lupinus angustifolius*)	gi|980951555	69366/5.96	17	417	SNLRLLGFGI; NADENQRNFL; AGSEDNVIRQ; LSEGDIFVIP; LDTEVKGLTF; INNPGNFYDF; AGHPISINAS; PGSTEDVERL
101	β-Conglutin (*Lupinus angustifolius*)	gi|169950562	69366/5.96	16	385	YPSSSKDQQS; SNKFGNFYEI; SNLRLLGFGI; IVRVSKEQVQ; TPNRNPQAQD; NADENQRNFL; GIRDQQRQQD; ELRKYAQSSS; LSEGDIFVIP; INNPGNFYDF; AGHPISINAS; PGSTEDVERL
102	β-Conglutin (*Lupinus angustifolius*)	gi|169950562	71883/5.82	6	154	RGQEQSHQDE; FPGSIEDVER; GVIVRVSKKQ; INANENQRNF; LAGSEDNVIK
103	β-Conglutin (*Lupinus angustifolius*)	gi|980951555	69366/5.96	14	408	YPSSSKDQQS; SNLRLLGFGI; YFSGFSRNTL; NADENQRNFL; EATFNTRYEE; INNPGNFYDF; EQEDDEQRHG; PGSTEDVERL
104	β-Conglutin (*Lupinus angustifolius*)	gi|980951550	68139/5.71	30	895	DALRLPAGTT; YFSGFSKNTL; NADENQRNFL; NAQPQQQQQR; RNPYYFSSER; EATFNTRYEE; AGSEDNVIRQ; INNPGNFYDF; PGSAEDVERL; YPSSSKDQQS; IKNQQQSYFA
105	β-Conglutin (*Lupinus angustifolius*)	gi|980951555	69366/5.96	26	834	KRQSYNLENG; YPSSSKDQQS; QEQSHQDEGV; SNKFGNFYEI; SNLRLLGFGI; GRREEEEEWQ; DALRLPAGTT; YFSGFSRNTL; IVRVSKEQVQ; TPNRNPQAQD; NADENQRNFL; PRRQRPQSRR; RNPYYFSSER; EATFNTRYEE; AGSEDNVIRQ; FQTLYRNRNG; NLRVVKLAIP; IQRILLGNED; LSEGDIFVIP; INNPGNFYDF; EQEDDEQRHG; AGHPISINAS
106	β-Conglutin (*Lupinus angustifolius*)	gi|980951550	68139/5.71	15	254	RNPYYFSSER; NADENQRNFL; AGSEDNVIRQ; INNPGNFYDF; YPSSSKDQQS; SNLRLLGFGI
107	α-Conglutin (*Lupinus angustifolius*)	gi|1117484096	74150/5.12	15	378	WFQLSADYVN; AEVLANAFGL; ETICTARLLE; LVAINLLDTT; NIAKPSRADL; SLLNQLDPSP; YNPNAGRISS; FKTNDLAATS; VNSLTLPILR; PVKQVFRGIP
108	α-Conglutin (*Lupinus angustifolius*)	gi|1117523166	57758/5.26	7	89	CQFQRLNALE; PILRWLGLAA; SSIRALPLDV; EHGSIYKNAM; VAHAFNLDRD
109	β-Conglutin (*Lupinus angustifolius*)	gi|980951548	70658/5.26	10	237	RRQRNPYYFS; NTLEATFNTR; HSDADYILVV; YEEIQRIILG; DNVIRQLDKE; LNGRATITIV; SGPFNLRSDE
110	β-Conglutin (*Lupinus angustifolius*)	gi|980951548	70658/5.64	18	416	FDQRTNRLEN; NPDKRQAYNL; YDFYPSSTKD; SKPNTLILPK; NTLEATFNTR; YERFQTLYKN; HSDADYILVV; LNGRATITIV; AIPINNPGNF
111	β-Conglutin (*Lupinus angustifolius*)	gi|1117364351	75163/5.75	29	775	EREQEQQPQY; LQNYRIVEFQ; EYGDALRLPA; EGVIVRVSKE; LLGFGINADE; GRRHEEEEKG; HRLRNPYYFS; SRPNTLILPK; GTTSYILNPD; NTLEATFNTH; QQDEQEVEEV; NQRNFLAGSE; SERFQTRYKN; HSDADYILVV; DNQDLRVVKL; YEEIQRILLG; RSYNARLSEG; DNVIRQLDRE; LNGRATITIV; AIPINNPGKF; SGPFNLRSNE; VKELIFPGSA; FDQRTNRLEN; NPDKRQAYNL; YDFYPSRTKD; RREQEQSHQD; PIYSNKFGNF; EDVERLIRNQ
112	β-Conglutin (*Lupinus angustifolius*)	gi|1117364351	75163/5.75	25	763	LQNYRIVEFQ; EYGDALRLPA; QQSYFSGFSK; EGVIVRVSKE; YEITPDRNPQ; LLGFGINADE; HRLRNPYYFS; VQDLDISLIF; QQDEQEVEEV; NQRNFLAGSE; SERFQTRYKN; HSDADYILVV; DNQDLRVVKL; TEISEGALLL; RSYNARLSEG; DNVIRQLDRE; LNGRATITIV; AIPINNPGKF; PHYNSKAIFV; VKELIFPGSA; FDQRTNRLEN; NPDKRQAYNL; YDFYPSRTKD; RREQEQSHQD; EDVERLIRNQ
113	β-Conglutin (*Lupinus angustifolius*)	gi|1117364351	75163/5.75	28	703	LQNYRIVEFQ; QQSYFSGFSK; EGVIVRVSKE; YEITPDRNPQ; LLGFGINADE; SRPNTLILPK; NTLEATFNTH; VQDLDISLIF; NQRNFLAGSE; DNQDLRVVKL; YEEIQRILLG; SSSRKGKPSE; TEISEGALLL; DNVIRQLDRE; LNGRATITIV; AIPINNPGKF; SGPFNLRSNE; PHYNSKAIFV; VKELIFPGSA; FDQRTNRLEN; NPDKRQAYNL; YDFYPSRTKD; RREQEQSHQD; PIYSNKFGNF; EDVERLIRNQ
114	β-Conglutin (*Lupinus angustifolius*)	gi|1117364351	75163/5.75	25	615	LQNYRIVEFQ; EYGDALRLPA; LLGFGINADE; HRLRNPYYFS; SRPNTLILPK; GTTSYILNPD; NQRNFLAGSE; SERFQTRYKN; HSDADYILVV; DNQDLRVVKL; YEEIQRILLG; SSSRKGKPSE; DNVIRQLDRE; LNGRATITIV; AIPINNPGKF; YEDEQEDEEQ; SGPFNLRSNE; VKELIFPGSA; FDQRTNRLEN; NPDKRQAYNL; YDFYPSRTKD; RREQEQSHQD; EDVERLIRNQ
115	α-Conglutin (*Lupinus angustifolius*)	gi|1117523166	57758/5.26	8	116	RLKTLTSLDF; PQIAALAGLT; PILRWLGLAA; SSIRALPLDV; NGLEETLCTL; KLRHNIGQST; SYVAFKTNDI

116	α-Conglutin (*Lupinus angustifolius*)	gi|1117484096	74150/5.12	4	73	AEVLANAFGL; YNPNAGRISS; VNSLTLPILR; PVKQVFRGIP
117	α-Conglutin (*Lupinus angustifolius*)	gi|1117523166	57758/5.26	3	58	SSIRALPLDV; PQIAALAGLT; SYVAFKTNDI
118	β-Conglutin (*Lupinus angustifolius*)	gi|980951555	69366/5.96	27	612	RTDRLENLQN; YPSSSKDQQS; QEQSHQDEGV; SNKFGNFYEI; YRIVEFQSKP; YFSGFSRNTL; IVRVSKEQVQ; TPNRNPQAQD; NADENQRNFL; NTLILPKHSD; EATFNTRYEE; ELRKYAQSSS; FQTLYRNRNG; AGSEDNVIRQ; NLRVVKLAIP; LSEGDIFVIP; LDTEVKGLTF; INNPGNFYDF; EQEDDEQRHG; AGHPISINAS; PGSTEDVERL
119	α-Conglutin (*Lupinus angustifolius*)	gi|1117484096	74150/5.12	7	248	AEVLANAFGL; ETICTARLLE; NIAKPSRADL; YNPNAGRISS; VNSLTLPILR; PVKQVFRGIP
120	β-Conglutin (*Lupinus angustifolius)*	gi|980951555	69366/5.96	27	742	QEQSHQDEGV; SNKFGNFYEI; DEGEGNYELV; IKNQQQSYFA; IVRVSKEQVQ; TPNRNPQAQD; NADENQRNFL; NAQPQQQQQR; LDISLTFIEI; AGSEDNVIRQ; NEGALLLPHY; LDTEVKGLTF; FNLRSNKPIY; NSKAIFVVLV; PGSTEDVERL
121	β-Conglutin (*Lupinus angustifolius*)	gi|169950562	71883/5.82	26	590	GPFNLRSNKP; KLSKGDVFII; VVDEGEGNYE; FPGSIEDVER; QGDALRLPAG; LVGIRDQQRQ; LIKNQQQSYF; TTSYILNPDD; TLEATFNTRY; QDEQEEEYEQ; ANAQPQQQQQ; LAGSEDNVIK; GEEEVRRYSD; LAGSEDNVIK; REKEGRRGRR
122	β-Conglutin (*Lupinus angustifolius*)	gi|169950562	71883/5.82	24	651	GPFNLRSNKP; HYNSKAIFIV; KLSKGDVFII; RGQEQSHQDE; VVDEGEGNYE; FPGSIEDVER; GVIVRVSKKQ; LIKNQQQSYF; IQELRKHAQS; INANENQRNF; ANAQPQQQQQ; SSGEGKPSES; LAGSEDNVIK; REKEGRRGRR
123	β-Conglutin (*Lupinus angustifolius*)	gi|169950562	71883/5.82	23	642	NGRATITIVN; GPFNLRSNKP; NQRTNRLENL; PDKRQVYNLE; RGQEQSHQDE; FPGSIEDVER; GVIVRVSKKQ; SSNLRLLGFG; LIKNQQQSYF; TLEATFNTRY; IQELRKHAQS; INANENQRNF; ANAQPQQQQQ; SSGEGKPSES; LAGSEDNVIK
124	β-Conglutin (*Lupinus angustifolius*)	gi|980951548	70658/5.64	34	1073	NPDKRQAYNL; YDFYPSSTKD; EDVERLIKNQ; GRREEEEEWQ; LQNYRIVEFQ; QQSYFNGFSR; LLGFGINADE; SKPNTLILPK; NTLEATFNTR; QQDEQEQEEV; NQRNFLAGSE; HSDADYILVV; LNGRATITIV; AIPINNPGNF; NEDGQEDEEQ
125	β-Conglutin (*Lupinus angustifolius*)	gi|980951548	70658/5.64	37	982	FDQRTNRLEN; NPDKRQAYNL; YDFYPSSTKD; EDVERLIKNQ; LQNYRIVEFQ; EHGDALRLPA; QQSYFNGFSR; LLGFGINADE; RRQRNPYYFS; SKPNTLILPK; GTTSYILNPD; NTLEATFNTR; QIQELRKHAQ; QQDEQEQEEV; NQRNFLAGSE; RSESEESREE; YERFQTLYKN; HSDADYILVV; DNQNLRVVKL; SSSGKGKPSE; RRYNAKLSEG; DNVIRQLDKE; EREQRREPSR; LNGRATITIV; AIPINNPGNF; SGPFNLRSDE; VKQLTFPGSV
126	β-Conglutin (*Lupinus angustifolius*)	gi|980951548	70658/5.64	27	669	GREQEQQPQH; YDFYPSSTKD; PIYSNKFGNF; EDVERLIKNQ; GRREEEEEWQ; LQNYRIVEFQ; QQSYFNGFSR; LLGFGINADE; RRQRNPYYFS; SKPNTLILPK; NTLEATFNTR; QIQELRKHAQ; NQRNFLAGSE; YERFQTLYKN; HSDADYILVV; DNQNLRVVKL; SSSGKGKPSE; DNVIRQLDKE; EREQRREPSR; LNGRATITIV; AIPINNPGNF; SGPFNLRSDE; VKQLTFPGSV
127	β-Conglutin (*Lupinus angustifolius*)	gi|980951550	68139/5.71	25	605	IVRVSKEQVQ; GIRDQQRQQD; NADENQRNFL; NAQPQQQQQR; FQTLYRNRNG; RKGKPYESGP; LSEGDIFVIP; INNPGNFYDF; FNLRSNKPIY; AGHPISINAS; PGSAEDVERL; YPSSSKDQQS; SNLRLLGFGI; IKNQQQSYFA; SNKFGNFYEI
128	β-Conglutin (*Lupinus angustifolius*)	gi|980951555	69366/5.96	14	448	QEQSHQDEGV; SNKFGNFYEI; SNLRLLGFGI; IVRVSKEQVQ; TPNRNPQAQD; TPNRNPQAQD; EQEVRRYSAR; AGSEDNVIRQ; RKGKPSKSGP; FNLRSNKPIY
129	α-Conglutin (*Lupinus angustifolius*)	gi|1117385782	67340/5.31	11	116	GVTETWNSNK; NIDEDTVHKL; PELRCAGVAF; ECQLDRLNAL; QEGKNNILSG; EPDNRIESEG; FDPQFLSQAL; IPAEVLANAF
130	β-Conglutin (*Lupinus angustifolius*)	gi|980951555	69366/5.96	32	949	YPSSSKDQQS; QEQSHQDEGV; SNKFGNFYEI; IKNQQQSYFA; IVRVSKEQVQ; NADENQRNFL; NAQPQQQQQR; ELRKYAQSSS; EQEVRRYSAR; AGSEDNVIRQ; LDTEVKGLTF; INNPGNFYDF; FNLRSNKPIY; PGSTEDVERL

131	β-Conglutin (*Lupinus angustifolius*)	gi|980951548	70658/5.64	25	608	NPDKRQAYNL; YDFYPSSTKD; GRREEEEEWQ; LQNYRIVEFQ; EHGDALRLPA; QQSYFNGFSR; RRQRNPYYFS; SKPNTLILPK; GTTSYILNPD; EEREQEQGSS; YERFQTLYKN; HSDADYILVV; SSSGRQSGYE; LNGRATITIV; AIPINNPGNF
132	β-Conglutin (*Lupinus angustifolius*)	gi|980951548	70658/5.64	23	508	GREQEQQPQH; YDFYPSSTKD; PIYSNKFGNF; GRREEEEEWQ; QQSYFNGFSR; LLGFGINADE; RRQRNPYYFS; SKPNTLILPK; NTLEATFNTR; YERFQTLYKN; HSDADYILVV; LNGRATITIV; AIPINNPGNF; EREQRREPSR; SGPFNLRSDE
133	β-Conglutin (*Lupinus angustifolius*)	gi|980951565	70082/6.18	8	166	PIYSNKFGNF; GRREEEEEWQ; RRQRNPYYFS; EEREQEQGSS; SERFQTLYRN; SGPFNLRSNK; RREQREEREQ
134	β-Conglutin (*Lupinus angustifolius*)	gi|980951555	69366/5.96	46	1155	EREQEQQPQH; RREEREQEQE; RTDRLENLQN; KRQSYNLENG; YPSSSKDQQS; QEQSHQDEGV; SNKFGNFYEI; SNLRLLGFGI; GRREEEEEWQ; YRIVEFQSKP; PRRQRPQSRR; NTLILPKHSD; DALRLPAGTT; YFSGFSRNTL; IVRVSKEQVQ; SYILNPDDNQ; EATFNTRYEE; TPNRNPQAQD; GIRDQQRQQD; NADENQRNFL; ELRKYAQSSS; EQEVRRYSAR; AGSEDNVIRQ; FQTLYRNRNG; NLRVVKLAIP; IQRILLGNED; RKGKPSKSGP; LSEGDIFVIP; LDTEVKGLTF; SSSRRQSGYE; INNPGNFYDF; EQEDDEQRHG; FNLRSNKPIY; AGHPISINAS; PGSTEDVERL
135	β-Conglutin (*Lupinus angustifolius*)	gi|980951555	69366/5.96	32	919	RREEREQEQE; RTDRLENLQN; KRQSYNLENG; YPSSSKDQQS; SNKFGNFYEI; SNLRLLGFGI; GRREEEEEWQ; YRIVEFQSKP; DALRLPAGTT; YFSGFSRNTL; IVRVSKEQVQ; TPNRNPQAQD; NADENQRNFL; PRRQRPQSRR; RNPYYFSSER; SYILNPDDNQ; EATFNTRYEE; ELRKYAQSSS; AGSEDNVIRQ; FQTLYRNRNG; NLRVVKLAIP; LDTEVKGLTF; SSSRRQSGYE; INNPGNFYDF; FNLRSNKPIY; PGSTEDVERL
136	β-Conglutin (*Lupinus angustifolius*)	gi|980951555	69366/5.96	38	979	RTDRLENLQN; KRQSYNLENG; YPSSSKDQQS; QEQSHQDEGV; SNKFGNFYEI; SNLRLLGFGI; GRREEEEEWQ; YRIVEFQSKP; DALRLPAGTT; YFSGFSRNTL; IVRVSKEQVQ; NADENQRNFL; PRRQRPQSRR; RNPYYFSSER; NTLILPKHSD; SYILNPDDNQ; EATFNTRYEE; ELRKYAQSSS; AGSEDNVIRQ; FQTLYRNRNG; NLRVVKLAIP; RKGKPSKSGP; LSEGDIFVIP; LDTEVKGLTF; INNPGNFYDF; EQEDDEQRHG; FNLRSNKPIY; AGHPISINAS; PGSTEDVERL
137	β-Conglutin (*Lupinus angustifolius*)	gi|980951555	69366/5.96	41	1019	EREQEQQPQH; KRQSYNLENG; YPSSSKDQQS; SNKFGNFYEI; RTDRLENLQN; SNLRLLGFGI; GRREEEEEWQ; YRIVEFQSKP; DALRLPAGTT; YFSGFSRNTL; IVRVSKEQVQ; TPNRNPQAQD; NADENQRNFL; RNPYYFSSER; NTLILPKHSD; SYILNPDDNQ; EATFNTRYEE; ELRKYAQSSS; AGSEDNVIRQ; FQTLYRNRNG; NLRVVKLAIP; IQRILLGNED; RKGKPSKSGP; LSEGDIFVIP; LDTEVKGLTF; INNPGNFYDF; EQEDDEQRHG; AGHPISINAS; FNLRSNKPIY; PGSTEDVERL
138	α-Conglutin (*Lupinus angustifolius*)	gi|1117385770	67340/5.31	18	67340	GVTETWNSNK; NIDEDTVHKL; PELRCAGVAF; QNPNERIKQI; EGQEEEETTT; IRVEEGLGVI; TTEERRRRRG; ECQLDRLNAL; QEGKNNILSG; SPKWQEQEEE; EPDNRIESEG; FDPQFLSQAL; EEEKEEPRQR
139	β-Conglutin (*Lupinus angustifolius*)	gi|1117364351	75163/5.75	19	415	QQSYFSGFSK; LLGFGINADE; NTLEATFNTH; QQDEQEVEEV; NQRNFLAGSE; DNVIRQLDRE; AIPINNPGKF; DILVIPAGHP; YDFYPSRTKD; PIYSNKFGNF
140	β-Conglutin (*Lupinus angustifolius*)	gi|1117364351	75163/5.75	19	532	LQNYRIVEFQ; LLGFGINADE; HRLRNPYYFS; NTLEATFNTH; HSDADYILVV; LNGRATITIV; SGPFNLRSNE; NPDKRQAYNL; PIYSNKFGNF
141	β-Conglutin (*Lupinus angustifolius*)	gi|980951548	70658/5.64	31	377	FDQRTNRLEN; NPDKRQAYNL; YDFYPSSTKD; ISINASSNLR; LQNYRIVEFQ; EHGDALRLPA; LLGFGINADE; QQSYFANAQP; SKPNTLILPK; GTTSYILNPD; NTLEATFNTR; NQRNFLAGSE
142	α-Conglutin (*Lupinus angustifolius*)	gi|1117484096	74150/5.12	8	79	QAFNVDEEII; LVAINLLDTT; SLLNQLDPSP; LSGFDPQFLT; CQLDRLNALE; RRFYIAGNPE; EQEEEGKNNV
143	α-Conglutin (*Lupinus angustifolius*)	gi|1117385770	67340/5.31	7	156	NIDEDTVHKL; QEGKNNILSG; EEEEVEEERG; FDPQFLSQAL; EPDNRIESEG; RESRRHRGGH
144	α-Conglutin (*Lupinus angustifolius*)	gi|1117385770	67340/5.31	6	102	NIDEDTVHKL; QEGKNNILSG; IRVEEGLGVI; FDPQFLSQAL; EPDNRIESEG; RESRRHRGGH

145	β-Conglutin (*Lupinus angustifolius*)	gi|980951548	70658/5.64	6	153	GREQEQQPQH; FDQRTNRLEN; NPDKRQAYNL; GRREEEEEWQ; LQNYRIVEFQ; SSSGKGKPSE; SGPFNLRSDE; LNGRATITIV
146	β-Conglutin (*Lupinus angustifolius*)	gi|980951548	70658/5.64	6	141	LLGFGINADE; NTLEATFNTR; HSDADYILVV; LNGRATITIV; NQRNFLAGSE
147	hypothetical protein Tanjilg (*Lupinus angustifolius*)	gi|1117500006	38596/6.47	66	1259	ITTDYMTYMF; PWAESGAEII; KTVDGPSSKD; PTVDVSVVDL; FIGDNRSSIF; KYDSVHGQWK; VESTGVFTDK; WRGGRAASFN; TVRLEKAATY; DAKAGIALNE; DKAAAHLKGG; CLAPLAKVIN; IIPSSTGAAK; DEIKKAIKEE; KYVKLVSWYD; VARVALQRDD; TLLFGEKSVA; AKKVIISAPS; DRFGIVEGLM; AVGKVLPVLN; SEGKLKGILG; NEWGYSTRVV; VELVAVNDPF; VYGHRNPEEI; KDAPMFVVGV; TTVHSITATQ; GKLTGMAFRV; YTEDDVVSTD; DLIAHVAKTL
148	hypothetical protein Tanjilg (*Lupinus angustifolius*)	gi|1117433037	46631/6.40	26	756	KTKEYAGDAA; ARESAGDAAQ; DVASGAGQYS; ESWTGWAKEK; DTIASNLEAA; LSEGLGLKND; TGTSGSAKDK; SNEELNWAKE
149	β-Conglutin (*Lupinus angustifolius*)	gi|149208403	54267/6.27	8	138	NQRTNRLENL; QNYRIIEFQS; QSYFSGFSKN; RQRNPYHFSS; NRFQTYYRNR; EEIERVLLGD
150	α-Conglutin (*Lupinus angustifolius*)	gi|1117484096	74150/5.12	3	59	LVAINLLDTT; SLLNQLDPSP; RRFYIAGNPE
151	α-Conglutin (*Lupinus angustifolius*)	gi|1117385770	67340/5.31	5	71	NIDEDTVHKL; QEGKNNILSG; ECQLDRLNAL; QEGKNNILSG; EPDNRIESEG; FDPQFLSQAL
152	α-Conglutin (*Lupinus angustifolius*)	gi|1117484096	74150/5.12	59	3	LVAINLLDTT; SLLNQLDPSP; RRFYIAGNPE
153	β-Conglutin (*Lupinus angustifolius*)	gi|1117364351	75163/5.75	21	615	LQNYRIVEFQ; EYGDALRLPA; QQSYFSGFSK; SRPNTLILPK; SRPNTLILPK; GTTSYILNPD; NTLEATFNTH; FDQRTNRLEN; NPDKRQAYNL; YDFYPSRTKD; KNGQIRVLER; AIPINNPGKF
154	β-Conglutin (*Lupinus angustifolius*)	gi|169950562	71883/5.82	6	158	FPGSIEDVER; INANENQRNF; LAGSEDNVIK; QLDREVKELT; SSNLRLLGFG
155	β-Conglutin (*Lupinus angustifolius*)	gi|980951555	69366/5.96	10	205	RTDRLENLQN; IKNQQQSYFA; NAQPQQQQQR; AGSEDNVIRQ; FQTLYRNRNG; PGSTEDVERL; LDTEVKGLTF; ELRKYAQSSS; IVRVSKEQVQ
156	α-Conglutin (*Lupinus angustifolius*)	gi|1117523166	57758/5.26	8	160	PQIAALAGLT; SSIRALPLDV; VAHAFNLDRD; VAIKSLDDNF; SYVAFKTNDI
157	β-Conglutin (*Lupinus angustifolius*)	gi|980951550	68139/5.71	6	131	YFSGFSKNTL; EATFNTRYEE; FQTLYRNRNG; NLRVVKLAIP; INNPGNFYDF; YPSSSKDQQS
158	Lupan putative TAG factor protein Tanjil (*Lupinus angustifolius*)	gi|1117470192	32193/6.33	41	649	MASSEQK**FPP; GDSGIGR**AVC; RLERVFR**TNI; TKGAIVAFTR; DVPMK**RPGQP; **FSYFFMTR**HA; **ALALQLVNK**G; IFTYVK**GHED;** IR**VNGVAPGP; KDAKDTLELI; INTTSVNAYK; IWTPLIPSSF; GHPSLLDYTS; KEEETAQFGS**
159	α -Conglutin (*Lupinus angustifolius*)	gi|980951550	68139/5.71	2	90	IVRVSKEQVQ; GIRDQQRQQD; ELRKYAQSSS; EQEVRRYSAR
160	β-Conglutin (*Lupinus angustifolius*)	gi|980951555	69366/5.96	20	477	YPSSSKDQQS; QEQSHQDEGV; SNKFGNFYEI; SNLRLLGFGI; NADENQRNFL; PRRQRPQSRR; RNPYYFSSER; EATFNTRYEE; FQTLYRNRNG; NLRVVKLAIP; LDTEVKGLTF; INNPGNFYDF; FNLRSNKPIY; PGSTEDVERL
161	β-Conglutin (*Lupinus angustifolius*)	gi|980951548	70658/5.64	9	134	NPDKRQAYNL; YDFYPSSTKD; LQNYRIVEFQ; SKPNTLILPK; DNVIRQLDKE; LNGRATITIV; AIPINNPGNF
162	Lupan putative TAG factor protein Tanjil (*Lupinus angustifolius*)	gi|1117470192	32193/6.33	14	154	MSRGSGGGYD; FRHKWGYEMP; RHITIFSPEG; LDQTSVSHLF; VDILARWIAD; PITKYLGLLA
163	β-Conglutin (*Lupinus angustifolius*)	gi|980951555	69366/5.96	9	138	RTDRLENLQN; YPSSSKDQQS; YRIVEFQSKP; YFSGFSRNTL; RNPYYFSSER; EATFNTRYEE; FQTLYRNRNG; INNPGNFYDF
164	β-Conglutin (*Lupinus angustifolius*)	gi|980951555	69366/5.96	6	102	RTDRLENLQN; KRQSYNLENG; YFSGFSRNTL; EATFNTRYEE; FQTLYRNRNG; YRIVEFQSKP

165	hypothetical protein Tanjilg (*Lupinus angustifolius)*	gi|1117568992	35865/5.96	43	635	AAAKLAAKDL; NEDIEVREHS; EDVNRAREQY; PNYTTHRYKD; KAGEYKDYTV; KDIGDVNRER; GLGAGEHEQK; TSENTGSKVG; KDSAADAAKR; EREQGFNLNS; EYTDYAAQKT; MNKAGEYTDY
166	β-Conglutin (*Lupinus angustifolius*)	gi|980951548	70658/5.64	25	568	NPDKRQAYNL; YDFYPSSTKD; LQNYRIVEFQ; SKPNTLILPK; NTLEATFNTR; NQRNFLAGSE; YERFQTLYKN; HSDADYILVV; SSSGKGKPSE; LNGRATITIV; AIPINNPGNF; NEDGQEDEEQ
167	β-Conglutin (*Lupinus angustifolius*)	gi|980951550	68139/5.71	45	953	EEEEEWQPRR; YRIVEFQSKP; DALRLPAGTT; YFSGFSKNTL; IVRVSKEQVQ; GIRDQQRQQD; NADENQRNFL; NAQPQQQQQR; RNPYYFSSER; NTLILPKHSD; EATFNTRYEE; ELRKYAQSSS; EQEVRRYSAR; AGSEDNVIRQ; FQTLYRNRNG; NLRVVKLAIP; IQSILLGNED; RKGKPYESGP; LSEGDIFVIP; LDREVKGLIF; RRQSGYERRE; INNPGNFYDF; EQEDDEQWHG; FNLRSNKPIY; AGHPISINAS; PGSAEDVERL; QEQQPQHGRR; RTNRLENLQN; KRQSYNLENG; YPSSSKDQQS; QEQSHQDEGV; SNKFGNFYEI; SNLRLLGFGI; IKNQQQSYFA
168	β-Conglutin (*Lupinus angustifolius*)	gi|1117364351	75163/5.75	40	1126	LQNYRIVEFQ; EYGDALRLPA; QQSYFSGFSK; EGVIVRVSKE; LLGFGINADE; HRLRNPYYFS; GTTSYILNPD; NTLEATFNTH; QQDEQEVEEV; NQRNFLAGSE; HSDADYILVV; DNQDLRVVKL; YEEIQRILLG; SSSRKGKPSE; LNGRATITIV; AIPINNPGKF; YEDEQEDEEQ; SGPFNLRSNE; DILVIPAGHP; NPDKRQAYNL; YDFYPSRTKD; RREQEQSHQD; LSINASSNLR; EDVERLIRNQ
169	α-Conglutin (*Lupinus angustifolius*)	gi|1117484096	74150/5.12	9	67	AEVLANAFGL; VPQNFVVAHQ; LVAINLLDTT; AGDEGFEFIA; SLLNQLDPSP; FKTNDLAATS; RRFYIAGNPE; PVKQVFRGIP; DDLRRGQLLV
170	β-Conglutin (*Lupinus angustifolius*)	gi|1117364351	75163/5.75	33	1037	LQNYRIVEFQ; EYGDALRLPA; QQSYFSGFSK; EGVIVRVSKE; LLGFGINADE; HRLRNPYYFS; AIPINNPGKF; SRPNTLILPK; NTLEATFNTH; QQDEQEVEEV; HSDADYILVV; YEEIQRILLG; EDVERLIRNQ; NPDKRQAYNL; LSINASSNLR; YDFYPSRTKD; VKELIFPGSA; SGPFNLRSNE; LNGRATITIV; DILVIPAGHP
171	β-Conglutin (*Lupinus angustifolius*)	gi|1117364351	75163/5.75	37	913	LQNYRIVEFQ; EYGDALRLPA; EGVIVRVSKE; LLGFGINADE; QQSYFANAQP; HRLRNPYYFS; GTTSYILNPD; QQDEQEVEEV; NQRNFLAGSE; QQQQQQREKE; SERFQTRYKN; HSDADYILVV; DNQDLRVVKL; YEEIQRILLG; RSYNARLSEG; DNVIRQLDRE; LNGRATITIV; AIPINNPGKF; YEDEQEDEEQ; SGPFNLRSNE; DILVIPAGHP; VKELIFPGSA; NPDKRQAYNL; RREQEQSHQD; LSINASSNLR; EDVERLIRNQ
172	β-Conglutin (*Lupinus angustifolius*)	gi|1117364351	75163/5.75	23	729	LQNYRIVEFQ; EYGDALRLPA; EGVIVRVSKE; LLGFGINADE; HRLRNPYYFS; NTLEATFNTH; QQDEQEVEEV; NQRNFLAGSE; HSDADYILVV; YEEIQRILLG; LNGRATITIV; NPDKRQAYNL; YDFYPSRTKD; RREQEQSHQD; PIYSNKFGNF; EDVERLIRNQ
173	α-Conglutin (*Lupinus angustifolius*)	gi|1117523166	57758/5.26	6	59	RPFYTNAPQE; FYLSGNQEQE; IYIQQGRGIF; FLQYQQKEGG; DNQLDQIPRR
174	β-Conglutin (*Lupinus angustifolius*)	gi|980951555	75163/5.75	6	166	QQSYFSGFSK; LLGFGINADE; AIPINNPGKF; FDQRTNRLEN, LQNYRIVEFQ; DNQDLRVVKL
175	α-Conglutin (*Lupinus angustifolius*)	gi|1117484096	74150/5.12	5	118	AEVLANAFGL; LVAINLLDTT; SLLNQLDPSP; PVKQVFRGIP; RRFYIAGNPE
176	α-Conglutin (*Lupinus angustifolius*)	gi|1117484096	74150/5.12	9	136	QAFNVDEEII; AEVLANAFGL; RLNQVSQLKY; PVKQVFRGIP; LSGFDPQFLT; LVAINLLDTT; SLLNQLDPSP; EQEEEGKNNV
177	β-Conglutin (*Lupinus angustifolius*)	gi|980951555	69366/5.96	24	765	QEQSHQDEGV; SNKFGNFYEI; IKNQQQSYFA; IVRVSKEQVQ; NADENQRNFL; EATFNTRYEE; ELRKYAQSSS; AGSEDNVIRQ; LDTEVKGLTF; PGSTEDVERL; FNLRSNKPIY; EQEDDEQRHG
178	β-Conglutin (*Lupinus angustifolius*)	gi|980951555	69366/5.96	32	864	QEQSHQDEGV; SNKFGNFYEI; DEGEGNYELV; IVRVSKEQVQ; GIRDQQRQQD; AGSEDNVIRQ; NAQPQQQQQR; ELRKYAQSSS; EQEVRRYSAR; LDTEVKGLTF; RKGKPSKSGP; PGSTEDVERL
179	β-Conglutin (*Lupinus angustifolius*)	gi|980951555	69366/5.96	35	924	YPSSSKDQQS; QEQSHQDEGV; SNKFGNFYEI; IKNQQQSYFA; RNPYYFSSER; ELRKYAQSSS; GRREEEEEWQ; YRIVEFQSKP; IVRVSKEQVQ; AGSEDNVIRQ; LDTEVKGLTF; FNLRSNKPIY

180	β-Conglutin (*Lupinus angustifolius*)	gi|980951550	68139/5.71	25	663	EEEEEWQPRR; YRIVEFQSKP; NADENQRNFL; NAQPQQQQQR; NTLILPKHSD; EATFNTRYEE; EQEVRRYSAR; AGSEDNVIRQ; RKGKPYESGP; INNPGNFYDF; FNLRSNKPIY; PGSAEDVERL; YPSSSKDQQS; SNKFGNFYEI; IKNQQQSYFA
181	β-Conglutin (*Lupinus angustifolius*)	gi|980951550	68139/5.71	31	856	EEEEEWQPRR; YRIVEFQSKP; DALRLPAGTT; YFSGFSKNTL; RNPYYFSSER; SYILNPDDNQ; EATFNTRYEE; AGSEDNVIRQ; INNPGNFYDF; FNLRSNKPIY; PGSAEDVERL; QEQQPQHGRR
182	β-Conglutin (*Lupinus angustifolius*)	gi|169950562	71883/5.82	26	794	GPFNLRSNKP; RGQEQSHQDE; FPGSIEDVER; QSYFSGFSKN; GVIVRVSKKQ; LIKNQQQSYF; TLEATFNTRY; IQELRKHAQS; INANENQRNF; ANAQPQQQQQ; SSGEGKPSES; LAGSEDNVIK; REKEGRRGRR
183	β-Conglutin (*Lupinus angustifolius*)	gi|169950562	71883/5.82	25	692	NGRATITIVN; RGQEQSHQDE; VVDEGEGNYE; FPGSIEDVER; QSYFSGFSKN; LVGIRDQQRQ; TLEATFNTRY; INANENQRNF; ANAQPQQQQQ
184	β-Conglutin (*Lupinus angustifolius*)	gi|980951555	69366/5.96	25	671	RTDRLENLQN; KRQSYNLENG; YPSSSKDQQS; YRIVEFQSKP; DALRLPAGTT; YFSGFSRNTL; RNPYYFSSER; SYILNPDDNQ; INNPGNFYDF
185	α-Conglutin (*Lupinus angustifolius*)	gi|1117385770	67340/5.31	18	248	GVTETWNSNK; NIDEDTVHKL; PELRCAGVAF; QNPNERIKQI; EGQEEEETTT; IRVEEGLGVI; TTEERRRRRG; ECQLDRLNAL; QEGKNNILSG; SPKWQEQEEE; EPDNRIESEG; FDPQFLSQAL; EEEKEEPRQR
186	β-Conglutin (*Lupinus angustifolius*)	gi|980951548	70658/5.64	29	871	NPDKRQAYNL; YDFYPSSTKD; LQNYRIVEFQ; EHGDALRLPA; QQSYFNGFSR; LLGFGINADE; GTTSYILNPD; NTLEATFNTR; NQRNFLAGSE
187	β-Conglutin (*Lupinus angustifolius*)	gi|980951548	70658/5.64	10	161	FDQRTNRLEN; YDFYPSSTKD; LLGFGINADE; QQSYFANAQP; QQQQQREKEG; AIPINNPGNF
188	β-Conglutin (*Lupinus angustifolius*)	gi|980951555	69366/5.96	21	523	RTDRLENLQN; KRQSYNLENG; YRIVEFQSKP; DALRLPAGTT; NADENQRNFL; AGSEDNVIRQ; RNPYYFSSER; SYILNPDDNQ; FQTLYRNRNG
189	β-Conglutin (*Lupinus angustifolius*)	gi|980951550	68139/5.71	33	954	EEEEEWQPRR; DALRLPAGTT; YFSGFSKNTL; NADENQRNFL; RNPYYFSSER; SYILNPDDNQ; EQEVRRYSAR; AGSEDNVIRQ; INNPGNFYDF
190	β-Conglutin (*Lupinus angustifolius*)	gi|980951548	70658/5.64	42	1068	GRREEEEEWQ; EHGDALRLPA; QQSYFNGFSR; SKPNTLILPK; GTTSYILNPD; NTLEATFNTR; YERFQTLYKN; HSDADYILVV; DNVIRQLDKE; LNGRATITIV; AIPINNPGNF; SGPFNLRSDE
191	β-Conglutin (*Lupinus angustifolius*)	gi|980951548	70658/5.64	37	1103	GREQEQQPQH; NPDKRQAYNL; YDFYPSSTKD; PIYSNKFGNF; EDVERLIKNQ; FDQRTNRLEN; GRREEEEEWQ; LQNYRIVEFQ; EHGDALRLPA; QQSYFNGFSR; LLGFGINADE; RRQRNPYYFS; NTLEATFNTR; QQDEQEQEEV; NQRNFLAGSE; YERFQTLYKN; HSDADYILVV; DNVIRQLDKE; VKQLTFPGSV; SGPFNLRSDE; AIPINNPGNF; LNGRATITIV; SSSGRQSGYE
192	β-Conglutin (*Lupinus angustifolius*)	gi|980951555	69366/5.96	18	515	EREQEQQPQH; RTDRLENLQN; YRIVEFQSKP; GRREEEEEWQ; NADENQRNFL; EATFNTRYEE; EQEVRRYSAR; AGSEDNVIRQ
193	β-Conglutin (*Lupinus angustifolius*)	gi|980951548	70658/5.64	39	1216	FDQRTNRLEN; NPDKRQAYNL; YDFYPSSTKD; VVVDEGEGNY; EDVERLIKNQ; GRREEEEEWQ; LQNYRIVEFQ; EHGDALRLPA; QQSYFNGFSR; ELVGIRDQER; LLGFGINADE; QQSYFANAQP; SKPNTLILPK; NTLEATFNTR; NQRNFLAGSE; QQQQQREKEG; YERFQTLYKN; HSDADYILVV; LNGRATITIV; AIPINNPGNF; NEDGQEDEEQ; SGPFNLRSDE; VKQLTFPGSV
194	β-Conglutin (*Lupinus angustifolius*)	gi|1117364351	75163/5.75	20	612	EREQEQQPQY; QQSYFSGFSK; LLGFGINADE; HSDADYILVV; LNGRATITIV; SGPFNLRSNE; YDFYPSRTKD; RREQEQSHQD; PIYSNKFGNF
195	β-Conglutin (*Lupinus angustifolius*)	gi|1117364351	75163/5.75	28	714	EQEQGSSSES; YEITPDRNPQ; LLGFGINADE; VQDLDISLIF; QQDEQEVEEV; NQRNFLAGSE; TEISEGALLL; YEDEQEDEEQ; VKELIFPGSA; RREQEQSHQD; EDVERLIRNQ
196	β-Conglutin (*Lupinus angustifolius*)	gi|1117364351	75163/5.75	16	530	EQEQGSSSES; QQSYFSGFSK; QQDEQEVEEV; NQRNFLAGSE; DNVIRQLDRE; VKELIFPGSA; RREQEQSHQD

197	β-Conglutin (*Lupinus angustifolius*)	gi|1117364351	75163/5.75	24	734	EYGDALRLPA; EGVIVRVSKE; LLGFGINADE; HRLRNPYYFS; QQDEQEVEEV; HSDADYILVV; LNGRATITIV; AIPINNPGKF; YEDEQEDEEQ; VKELIFPGSA
198	β-Conglutin (*Lupinus angustifolius*)	gi|1117364351	75163/5.75	31	821	EQEQGSSSES; QQSYFSGFSK; YEITPDRNPQ; LLGFGINADE; VQDLDISLIF; QQDEQEVEEV; TEISEGALLL; AIPINNPGKF; YEDEQEDEEQ; YEDEQEDEEQ; SGPFNLRSNE; NPDKRQAYNL
199	β-Conglutin (*Lupinus angustifolius*)	gi|980951550	68139/5.71	21	570	EEEEEWQPRR; YFSGFSKNTL; PGSAEDVERL; NADENQRNFL; NADENQRNFL; YPSSSKDQQS; AGSEDNVIRQ; INNPGNFYDF; PGSAEDVERL
200	β-Conglutin (*Lupinus angustifolius*)	gi|980951555	69366/5.96	14	370	RTDRLENLQN; NADENQRNFL; RNPYYFSSER; EATFNTRYEE; AGSEDNVIRQ; INNPGNFYDF
201	β-Conglutin (*Lupinus angustifolius*)	gi|980951550	68139/5.71	4	78	IVRVSKEQVQ; ELRKYAQSSS; INNPGNFYDF; YPSSSKDQQS
202	β-Conglutin (*Lupinus angustifolius*)	gi|149208403	54267/6.27	8	153	IPINNPGKLY; RQRNPYHFSS; RQRNPYHFSS; EEIERVLLGD; GVIVRVSKKQ; NQNLRVAKLA
203	α-Conglutin (*Lupinus angustifolius*)	gi|1117484096	74150/5.12	2	66	VNSLTLPILR; YNPNAGRISS
204	β-Conglutin (*Lupinus angustifolius*)	gi|98051548	70658/5.64	6	141	LLGFGINADE; NTLEATFNTR; HSDADYILVV; LNGRATITIV; NQRNFLAGSE
205	β-Conglutin (*Lupinus angustifolius*)	gi|149208403	54267/6.27	6	106	DFYPSTTKDQ; RQRNPYHFSS; NRFQTYYRNR; TLEATFNTRY; NRFQTYYRNR; IPINNPGKLY
206	β-Conglutin (*Lupinus angustifolius*)	gi|980951555	69366/5.96	11	200	RTDRLENLQN; QEQSHQDEGV; IVRVSKEQVQ; FQTLYRNRNG; INNPGNFYDF; LDTEVKGLTF; YPSSSKDQQS; IQRILLGNED; EQEDDEQRHG
207	α-Conglutin (*Lupinus angustifolius*)	gi|1117385770	67340/5.31	6	84	NIDEDTVHKL; IRVEEGLGVI; QEGKNNILSG; FDPQFLSQAL
208	β-Conglutin (*Lupinus angustifolius*)	gi|149208403	54267/6.27	4	126	RQRNPYHFSS; NRFQTYYRNR; EEIERVLLGD; TLEATFNTRY
209	β-Conglutin (*Lupinus angustifolius*)	gi|980951550	68139/5.71	10	215	EEEEEWQPRR; RNPYYFSSER; EATFNTRYEE; FQTLYRNRNG; INNPGNFYDF; YPSSSKDQQS; RTNRLENLQN; YPSSSKDQQS; QEQQPQHGRR
210	α-Conglutin (*Lupinus angustifolius*)	gi|1117433037	74150/5.12	4	117	AEVLANAFGL; VNSLTLPILR; YNPNAGRISS; PVKQVFRGIP
211	β-Conglutin (*Lupinus angustifolius*)	gi|149208403	54267/6.27	10	126	RGQEQSHQDE; RQRNPYHFSS; TLEATFNTRY; EEIERVLLGD; QNYRIIEFQS; NQRTNRLENL
212	β-Conglutin (*Lupinus angustifolius*)	gi|980951555	69366/5.96	20	493	QEQSHQDEGV; YPSSSKDQQS; NADENQRNFL; ELRKYAQSSS; LDTEVKGLTF; INNPGNFYDF; PGSTEDVERL; EQEDDEQRHG; YRIVEFQSKP
213	β-Conglutin (*Lupinus angustifolius*)	gi|980951555	69366/5.96	17	430	QEQSHQDEGV; SNLRLLGFGI; IVRVSKEQVQ; NADENQRNFL; ELRKYAQSSS; LSEGDIFVIP; LDTEVKGLTF; FNLRSNKPIY; AGHPISINAS; PGSTEDVERL
214	β-Conglutin (*Lupinus angustifolius*)	gi|980951555	69366/5.96	17	537	QEQSHQDEGV; SNKFGNFYEI; GRREEEEEWQ; IVRVSKEQVQ; NADENQRNFL; AGSEDNVIRQ; LDTEVKGLTF; FNLRSNKPIY; EQEVRRYSAR
215	β-Conglutin (*Lupinus angustifolius*)	gi|1117364351	75163/5.75	5	139	LLGFGINADE; QQDEQEVEEV; NQRNFLAGSE; DNVIRQLDRE
216	β-Conglutin (*Lupinus angustifolius*)	gi|169950562	71883/5.82	11	289	NGRATITIVN; GPFNLRSNKP; FPGSIEDVER; GVIVRVSKKQ; SSNLRLLGFG; INANENQRNF
217	α-Conglutin (*Lupinus angustifolius*)	gi|1117523166	57758/5.26	3	66	PQIAALAGLT; SSIRALPLDV; SYVAFKTNDI
218	β-Conglutin (*Lupinus angustifolius*)	gi|169950562	71883/5.82	23	708	GPFNLRSNKP; KLSKGDVFII; QLDREVKELT; RGQEQSHQDE; FPGSIEDVER; GVIVRVSKKQ; TTSYILNPDD; TLEATFNTRY; IQELRKHAQS; QDEQEEEYEQ; INANENQRNF; NQNLRVAKLA; SSGEGKPSES; GEEEVRRYSD; LAGSEDNVIK
219	β-Conglutin (*Lupinus angustifolius*)	gi|169950562	71883/5.82	13	335	GPFNLRSNKP; RGQEQSHQDE; FPGSIEDVER; QSYFSGFSKN; GVIVRVSKKQ; SSNLRLLGFG; INANENQRNF; INANENQRNF

220	β-Conglutin (*Lupinus angustifolius*)	gi|980951555	69366/5.96	16	388	RTDRLENLQN; KRQSYNLENG; SNKFGNFYEI; IKNQQQSYFA; NADENQRNFL; NAQPQQQQQR; EATFNTRYEE; AGSEDNVIRQ; PGSTEDVERL
221	β-Conglutin (*Lupinus angustifolius*)	gi|980951555	69366/5.96	32	949	RTDRLENLQN; YPSSSKDQQS; QEQSHQDEGV; GRREEEEEWQ; SNKFGNFYEI; SNLRLLGFGI; IKNQQQSYFA; YRIVEFQSKP; DALRLPAGTT; NADENQRNFL; NAQPQQQQQR; PRRQRPQSRR; SYILNPDDNQ; EQEVRRYSAR; ELRKYAQSSS; GIRDQQRQQD; IVRVSKEQVQ; TPNRNPQAQD; AGSEDNVIRQ; NLRVVKLAIP; RKGKPSKSGP; LDTEVKGLTF; EQEDDEQRHG; FNLRSNKPIY; PGSTEDVERL; INNPGNFYDF
222	β-Conglutin (*Lupinus angustifolius*)	gi|980951555	69366/5.96	37	1084	RTDRLENLQN; YPSSSKDQQS; QEQSHQDEGV; SNKFGNFYEI; DEGEGNYELV; SNLRLLGFGI; IKNQQQSYFA; GRREEEEEWQ; YRIVEFQSKP; YFSGFSRNTL; IVRVSKEQVQ; TPNRNPQAQD; GIRDQQRQQD; NADENQRNFL; NAQPQQQQQR; PRRQRPQSRR; RNPYYFSSER; NTLILPKHSD; EATFNTRYEE; ELRKYAQSSS; EQEVRRYSAR; AGSEDNVIRQ; NLRVVKLAIP; RKGKPSKSGP; LDTEVKGLTF; INNPGNFYDF; EQEDDEQRHG; FNLRSNKPIY; NSKAIFVVLV; PGSTEDVERL
223	β-Conglutin (*Lupinus angustifolius*)	gi|980951548	68139/5.71	31	856	GREQEQQPQH; FDQRTNRLEN; NPDKRQAYNL; YDFYPSSTKD; GRREEEEEWQ; LQNYRIVEFQ; EHGDALRLPA; QQSYFNGFSR; LLGFGINADE; PRRQRPQSRR; RRQRNPYYFS; SKPNTLILPK; GTTSYILNPD; NTLEATFNTR; NQRNFLAGSE; YERFQTLYKN; HSDADYILVV; DNQNLRVVKL; EREQRREPSR; LNGRATITIV; AIPINNPGNF
224	β-Conglutin (*Lupinus angustifolius*)	gi|980951550	68139/5.71	40	1060	EEEEEWQPRR; YRIVEFQSKP; DALRLPAGTT; YFSGFSKNTL; IVRVSKEQVQ; GIRDQQRQQD; NADENQRNFL; RNPYYFSSER; NTLILPKHSD; SYILNPDDNQ; EATFNTRYEE; ELRKYAQSSS; EQEVRRYSAR; AGSEDNVIRQ; FQTLYRNRNG; NLRVVKLAIP; RKGKPYESGP; LDREVKGLIF; QRREPRRERE; INNPGNFYDF; FNLRSNKPIY; NSKAIFVVVV; PGSAEDVERL; QEQQPQHGRR; RTNRLENLQN; KRQSYNLENG; YPSSSKDQQS; SNKFGNFYEI; DEGEGNYELV; SNLRLLGFGI
225	β-Conglutin (*Lupinus angustifolius*)	gi|980951555	69366/5.96	32	971	EREQEQQPQH; RTDRLENLQN; KRQSYNLENG; YPSSSKDQQS; SNKFGNFYEI; SNLRLLGFGI; GRREEEEEWQ; YRIVEFQSKP; DALRLPAGTT; YFSGFSRNTL; IVRVSKEQVQ; NADENQRNFL; PRRQRPQSRR; RNPYYFSSER; NTLILPKHSD; SYILNPDDNQ; EATFNTRYEE; ELRKYAQSSS; AGSEDNVIRQ; EEREQEQGSS; FQTLYRNRNG; NLRVVKLAIP; IQRILLGNED; SSSRRQSGYE; INNPGNFYDF; FNLRSNKPIY
226	β-Conglutin (*Lupinus angustifolius*)	gi|980951550	68139/5.71	40	1075	EEEEEWQPRR; YRIVEFQSKP; DALRLPAGTT; YFSGFSKNTL; IVRVSKEQVQ; GIRDQQRQQD; NADENQRNFL; QRPQSRREER; RNPYYFSSER; NTLILPKHSD; SYILNPDDNQ; EATFNTRYEE; ELRKYAQSSS; EQEVRRYSAR; AGSEDNVIRQ; FQTLYRNRNG; NLRVVKLAIP; RKGKPYESGP; LDREVKGLIF; INNPGNFYDF; FNLRSNKPIY; NSKAIFVVVV; PGSAEDVERL; QEQQPQHGRR; RTNRLENLQN; KRQSYNLENG; YPSSSKDQQS; SNKFGNFYEI; DEGEGNYELV; SNLRLLGFGI
227	β-Conglutin (*Lupinus angustifolius*)	gi|980951550	68139/5.71	31	781	EEEEEWQPRR; YRIVEFQSKP; DALRLPAGTT; YFSGFSKNTL; IVRVSKEQVQ; GIRDQQRQQD; NADENQRNFL; QRPQSRREER; RNPYYFSSER; NTLILPKHSD; SYILNPDDNQ; EATFNTRYEE; ELRKYAQSSS; EQEVRRYSAR; AGSEDNVIRQ; FQTLYRNRNG; NLRVVKLAIP; FNLRSNKPIY; NSKAIFVVVV; PGSAEDVERL; RTNRLENLQN; YPSSSKDQQS; SNKFGNFYEI; DEGEGNYELV; SNLRLLGFGI
228	β-Conglutin (*Lupinus angustifolius*)	gi|980951550	68139/5.71	36	835	EEEEEWQPRR; YRIVEFQSKP; DALRLPAGTT; YFSGFSKNTL; IVRVSKEQVQ; GIRDQQRQQD; NADENQRNFL; NAQPQQQQQR; SYILNPDDNQ; EATFNTRYEE; ELRKYAQSSS; EQEVRRYSAR; AGSEDNVIRQ; FQTLYRNRNG; NLRVVKLAIP; RKGKPYESGP; LDREVKGLIF; INNPGNFYDF; FNLRSNKPIY; NSKAIFVVVV; PGSAEDVERL; QEQQPQHGRR; RTNRLENLQN; KRQSYNLENG; YPSSSKDQQS; SNKFGNFYEI; DEGEGNYELV; SNLRLLGFGI; IKNQQQSYFA

229	β-Conglutin (*Lupinus angustifolius*)	gi|169950562	71883/5.82	29	958	NGRATITIVN; GPFNLRSNKP; KLSKGDVFII; QLDREVKELT; NQRTNRLENL; PDKRQVYNLE; DFYPSTTKDQ; RGQEQSHQDE; IYSNKFGNFY; FPGSIEDVER; QNYRIIEFQS; QSYFSGFSKN; GVIVRVSKKQ; LVGIRDQQRQ; SSNLRLLGFG; LIKNQQQSYF; TLEATFNTRY; IQELRKHAQS; QDEQEEEYEQ; INANENQRNF; ANAQPQQQQQ; SSGEGKPSES; GEEEVRRYSD; LAGSEDNVIK; REKEGRRGRR
230	β-Conglutin (*Lupinus angustifolius*)	gi|980951555	69366/5.96	35	823	EREQEQQPQH; RTDRLENLQN; YPSSSKDQQS; SNKFGNFYEI; DEGEGNYELV; SNLRLLGFGI; GRREEEEEWQ; YRIVEFQSKP; YFSGFSRNTL; IVRVSKEQVQ; GIRDQQRQQD; PRRQRPQSRR; NADENQRNFL; RNPYYFSSER; NTLILPKHSD; EATFNTRYEE; ELRKYAQSSS; AGSEDNVIRQ; LSEGDIFVIP; FQTLYRNRNG; FNLRSNKPIY
